# Accelerating Crystallization of Open Organic Materials by Poly(ionic liquid)s

**DOI:** 10.1002/anie.202008415

**Published:** 2020-10-04

**Authors:** Su‐Yun Zhang, Han Miao, He‐min Zhang, Jun‐Hao Zhou, Qiang Zhuang, Yu‐Jia Zeng, Zhiming Gao, Jiayin Yuan, Jian‐Ke Sun

**Affiliations:** ^1^ School of Chemistry and Chemical Engineering Beijing Institute of Technology Beijing P. R. China; ^2^ College of Physics and Optoelectronic Engineering Shenzhen University Shenzhen 518060 P. R. China; ^3^ School of Materials Science and Engineering Georgia Institute of Technology Atlanta GA 30332 USA; ^4^ School of Energy and Chemical Engineering Ulsan National Institute of Science & Technology (UNIST) Ulsan 689-798 Republic of Korea; ^5^ Department of Materials and Environmental Chemistry Stockholm University 10691 Stockholm Sweden; ^6^ School of Chemistry and Chemical Engineering Northwestern Polytechnical University Xi'an Shaanxi 710072 P. R. China

**Keywords:** crystallization, open organic materials, organic cages, poly(ionic liquid)s

## Abstract

The capability to significantly shorten the synthetic period of a broad spectrum of open organic materials presents an enticing prospect for materials processing and applications. Herein we discovered 1,2,4‐triazolium poly(ionic liquid)s (PILs) could serve as a universal additive to accelerate by at least one order of magnitude the growth rate of representative imine‐linked crystalline open organics, including organic cages, covalent organic frameworks (COFs), and macrocycles. This phenomenon results from the active C5‐protons in poly(1,2,4‐triazolium)s that catalyze the formation of imine bonds, and the simultaneous salting‐out effect (induced precipitation by decreasing solubility) that PILs exert on these crystallizing species.

## Graphical abstract

1,2,4‐Triazolium poly(ionic liquid) (PIL) is an additive to accelerate by at least one order of magnitude the crystallization rate of imine‐linked crystalline open organics (organic cages, covalent organic frameworks (COFs), and macrocycles). This acceleration effect stems from the active C5 protons in polytriazolium that catalyze formation of imine bonds and the salting‐out effect of PILs on these crystallizing species.

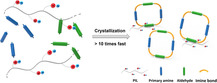

## Introduction

Recently, open organic compounds including covalent organic frameworks (COFs),^[1]^ organic cages,^[2]^ and macrocycles^[3]^ as emerging functional materials have been studied extensively because of a wide range of potential applications in gas storage,^[4]^ catalysis,^[5]^ energy storage,^[6]^ and light harvesting.^[7]^ Among diverse sorts of open organics, imine‐linked crystalline open materials, the monomers of which are organized in a crystalline state predictably using directional bonding principles, have drawn particular interest because of their excellent thermal, oxidative and hydrolytic stability, and ease of modification by a desired functionality.^[8]^ Imine‐linked open organic compounds are conventionally synthesized under conditions allowing for formation and exchange of imine bonds, which typically requires thermal treatment and a long reaction time ranging from several hours up to days owing to slow kinetics in nucleation and crystallization of these materials at room temperature. Efforts to speed up crystallization have been attempted, such as energy input (such as microwave synthesis) or adding CH_3_COOH/CF_3_COOH/Lewis acid as catalyst.^[9]^ Comparatively speaking, utilization of intrusive stimulus at room temperature is more desirable as a low‐energy, better controlled process,^[10]^ and in addition it avoids the conventional acid catalysts as additives that risk the stability of the imine bond. A challenge is how, in such a mild, environmentally benign route, to reduce the synthesis duration of open organics without compromising the quality of crystallinity and pore matrixes.

Poly(ionic liquid)s (PILs) carrying an ionic liquid moiety in their monomer unit have attracted wide interest across polymer and materials science owing to their broad physicochemical property window, unprecedentedly rich choices in repeating unit structure, and their multifunctional nature for materials design.^[11]^ Current advance in PIL chemistry and physics proceeds fast and has impacted a multiple of subfields of chemistry and materials science.^[12]^ Recently, the 1,2,4‐triazolium‐type PILs have served as polycarbene precursors through the deprotonation of C5‐proton in the triazolium ring. This decent chemistry was adopted to immobilize metal clusters of high catalytic activity and to sense protons at ultra‐low detection limit in solution.^[13]^

**Scheme 1:**
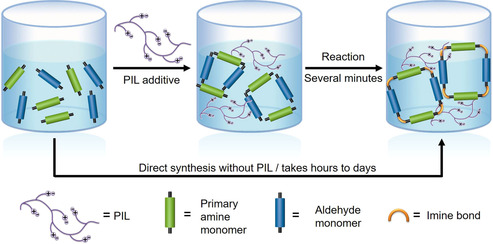
Representation of the accelerated room‐temperature crystallization of imine bond‐based open organics by PILs. Crystallization time is reduced from hours/days to several minutes by PIL additive.

Herein, we report our latest discovery that the 1,2,4‐triazolium PILs could serve as a macromolecular additive to cross the crystallization energy barrier at room temperature in imine‐linked crystalline open organics, including organic cages, COFs, and macrocycles (Scheme 1). As an obvious outcome, this discovery shortens the crystallization process to form open organics from hours/days to several minutes while maintaining the high quality of crystals. This acceleration effect stems from the active C5‐protons in polytriazoliums that catalyze the formation of imine bonds, plus the simultaneous salting‐out effect of PILs that exert on these crystals, an effect that has recently been reported.^[14]^ Note that the catalytic role of PILs in the crystallization process does not deteriorate the bulk properties of crystals, but only deposits a small amount of PIL chains on the crystal surface, which in fact facilitates further applications where easy solubilization and functionalization of crystals in liquid phases is needed.

## Results and Discussion

**Figure 1:**
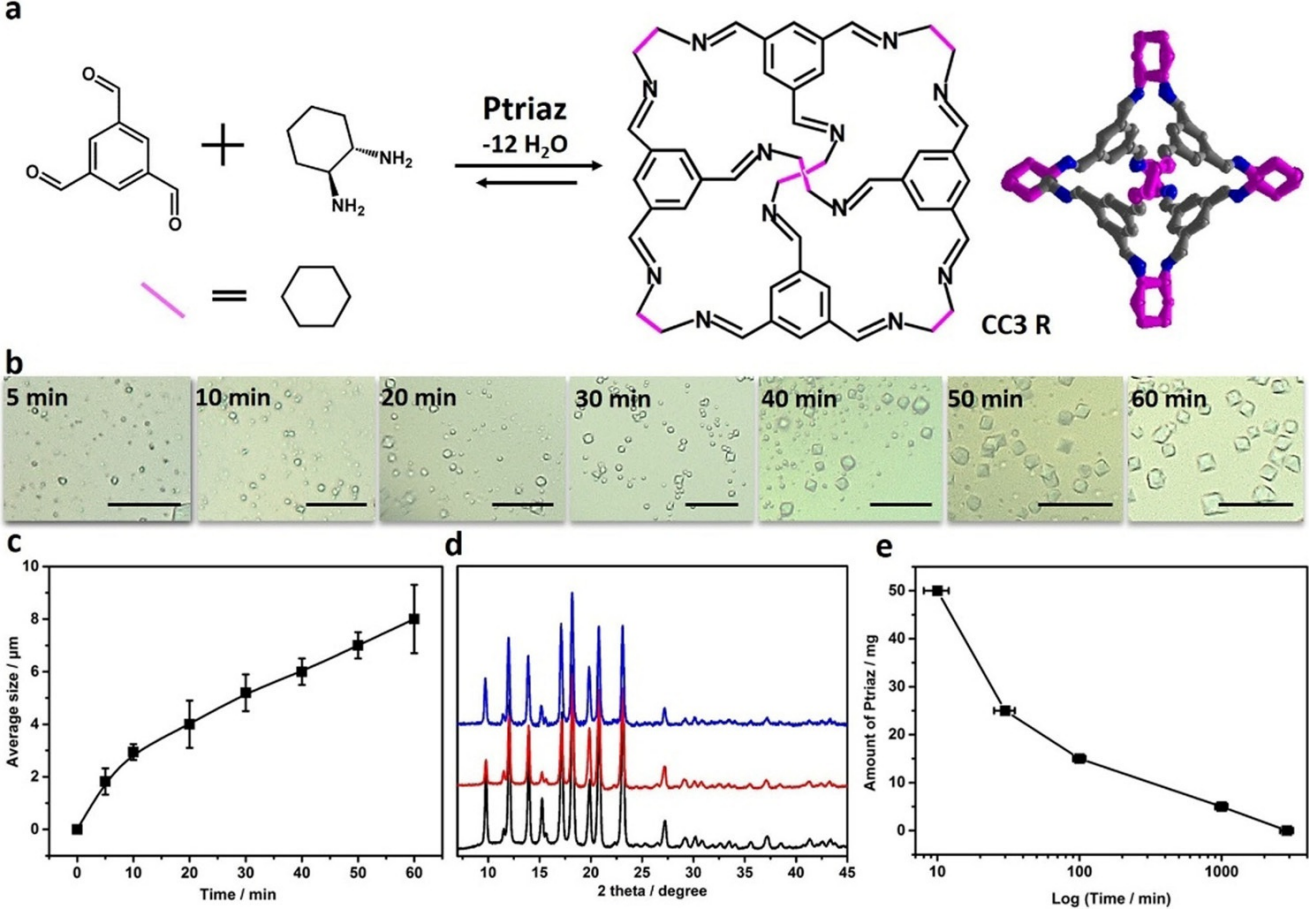
a) Synthesis of porous organic cage CC3R. b) Time‐dependent single crystals of CC3R optic images in the presence of Ptriaz, scale bar, 25 μm. c) Average sizes of CC3R single crystals increasing with time in the presence of Ptriaz. d) PXRD pattern of the as‐synthesized CC3R^2a^ re‐crystallized from chloroform (black), direct synthesis from chloroform without Ptraiz (red), synthesis from chloroform with Ptraiz additive (blue). e) The correlation of the Ptriaz concentration against crystallization time to grow the single crystals of the same average size (ca. 5 μm).

**Figure 2:**
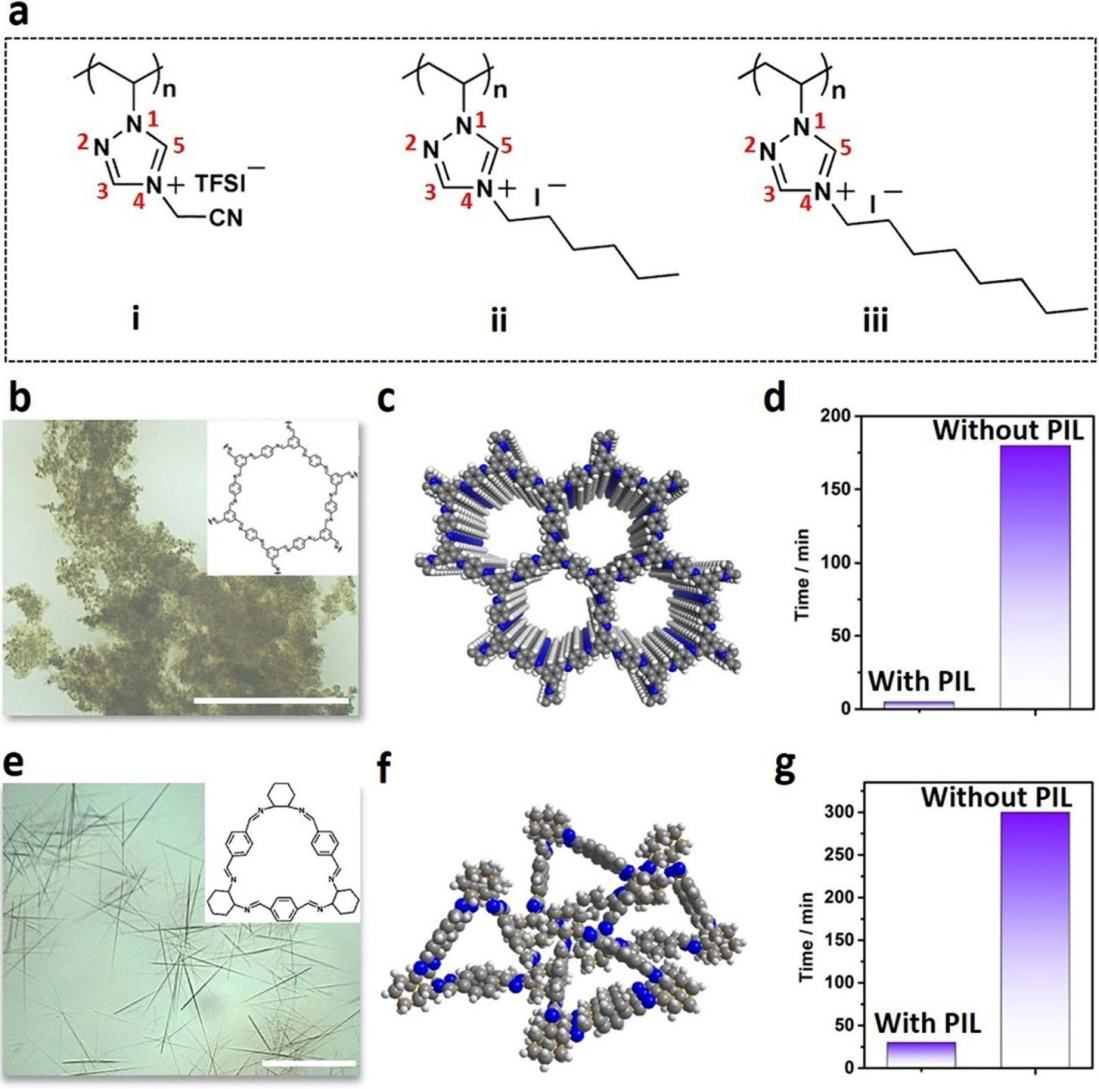
a) Various PILs used for accelerating the crystallization of open organics. The positions of atoms in the triazolium ring are numerically labeled. i) Poly(4‐cyanomethyl‐1‐vinyl‐1,2,4‐triazolium bis(trifluoromethane sulfonyl)imide), ii) poly(4‐hexyl‐1‐vinyl‐1,2,4‐triazolium iodide) (Ptriaz), iii) poly(4‐octyl‐1‐vinyl‐1,2,4‐triazolium iodide). The microscope image of crystals (crystallite for COFs, the TEM images are shown in the Supporting Information) and corresponding chemical structures growth with different PILs additive, b),c) BTA‐PED COF with poly(4‐cyanomethyl‐1‐vinyl‐1,2,4‐triazolium bis(trifluoromethane sulfonyl)imide); e),f) TPA‐DACH macrocycle with poly(4‐octyl‐1‐vinyl‐1,2,4‐triazolium iodide), scale bar: 100 μm. d),g) The comparison of crystallizing time for growth of similar sized crystallites (ca. 36 nm for BTA‐PED COF; Supporting Information, Figure S3) and crystals (ca. 80 μm for TPA‐DACH macrocycle; Supporting Information, Figure S4) with and without PILs.

This discovery starts with synthesis of CC3R, a prototypical porous organic [4+6] cycloimine. It was synthesized first by Cooper's group,^[2a]^ and is useful in fields of gas storage and separation, catalysis, and so on.^[15]^ The state‐of‐the‐art synthesis of CC3R at room temperature requires a prolonged time to yield well‐shaped micrometer‐sized single crystals in the presence of an acid (CF_3_COOH) to catalyze the imine bond formation. To speed up this synthesis, in our initial experiments CC3R was prepared at room temperature by condensation of 8 mg of (*R*,*R*)‐1,2‐diaminocyclohexane (DACH) and 8 mg of 1,3,5‐benzenetricarbaldehyde (BTA) in the presence of 25 mg of a 1,2,4‐triazolium PIL, poly(4‐hexyl‐1‐vinyl‐1,2,4‐triazolium iodide) (denoted as Ptriaz, Figure 2 a, ii) in 0.7 mL of chloroform (Figure 1 a). The reaction runs surprisingly as quickly as 10 s, accompanied by appearance of an optically turbid dispersion. Single crystals with an average size of 2 μm, as determined by optical microscopy (Figure 1 b), form within 5 min. The recorded X‐ray diffraction (XRD) pattern confirms the CC3R phase (Figure 1 d). By contrast, in a Ptriaz‐free control experiment crystal of a similar size formed only after about 36 h, that is, a slowing‐down factor of over 400 times. Moreover, even adding equimolar amounts of conventional acid CF_3_COOH (0.0052 mmol) instead of Ptriaz still took about 28 h to generate similarly sized CC3R crystals. When crystals were not isolated from the Ptriaz solution, they grew further from 2 to 8 μm within 60 min, as monitored by time dependent microscopy imaging (Figure 1 b, c; Supporting Information, Figure S1).

As expected, the crystallization rate is dependent on the concentration of PIL additive (Figure 1 e). The products obtained from experiments were denoted as CC3R‐Ptriaz‐*x*, where *x* is the amount of Ptriaz added in solution in mg for the same reaction. Raising Ptriaz from 25 mg (ca. 30 min) in our initial test to 50 mg, it takes about 10 min to generate averagely 5 μm sized single crystals, while 17 h at a PIL content of 5 mg (Figure 1 e), that is, a very sensitive reaction towards PIL as catalyst. The surface area of CC3R‐Ptriaz‐25 calculated from the Brunauer–Emmett–Teller (BET) equation (*S*_BET_) is 624 m^2^ g^−1^, comparable to CC3R that was synthesized without Ptriaz (*S*_BET_=559 m^2^ g^−1^; Supporting Information, Figure S2). It is clear that the addition of PILs to regulate the growth rate of crystals does not sacrifice their surface area that is important for surface‐involved applications, such as sorption or catalysis.

As the PIL chemistry allows for vast flexibility in chemical structural design (Figure 2 a) in terms of the type of backbone, alkyl spacer and anions, the current approach can be generalized by pairing different PILs with individual imine‐linked open organics in corresponding solvents. The tested open organics here include COFs (BTA‐PED COF,^[16]^ PED=1,4‐phenylenediamine), and organic macrocycles^[17]^ ([3+3] cyclocondensation of (*R*,*R*)‐1,2‐diaminocyclohexane with terephthalaldehyde (TPA), denoted as TPA‐DACH macrocycle) (Figure 2 b–g; Supporting Information, Figures S3, S4). Broadly speaking, the crystallization rate of all tests is accelerated by a factor of 10 to 36 folds in the presence of three employed PILs, Ptriaz, poly(4‐cyanomethyl‐1‐vinyl‐1, 2, 4‐triazolium bis(trifluoromethane sulfonyl)imide) (Figure 2 a, i), or poly(4‐octyl‐1‐vinyl‐1,2,4‐triazolium iodide) (Figure 2 a, iii). The crystalline phase was all evidenced by PXRD pattern (Supporting Information, Figure S5, S6).

The current method can be additionally extended to isolate the kinetic crystalline product over the thermodynamic one, while the conventional method unfavorably encounters their mixture that is hard to separate. One example illustrated here is the trianglimine macrocycle system, in which the kinetic product via [3+3] cyclocondensation reaction between (*R*,*R*)‐1,2‐diaminocyclohexane and isophthalaldehyde (IPA), is usually accompanied with a thermodynamically favored [2+2] cyclocondensation byproduct (a lower energy state) due to competition of the steric effect and conformational requirement (Supporting Information, Figure S7a),^[18]^ thus tedious purification is always necessary. Here, Ptriaz additive can successfully suppress the thermodynamic product and boost the formation of dynamically favored [3+3] cyclocondensation single crystals in 30 min (average sizes: 110±11.3 μm; Supporting Information, Figure S8) without further purification (^1^H NMR spectrometry measurements are shown in the Supporting Information, Figure S7b).

**Figure 3:**
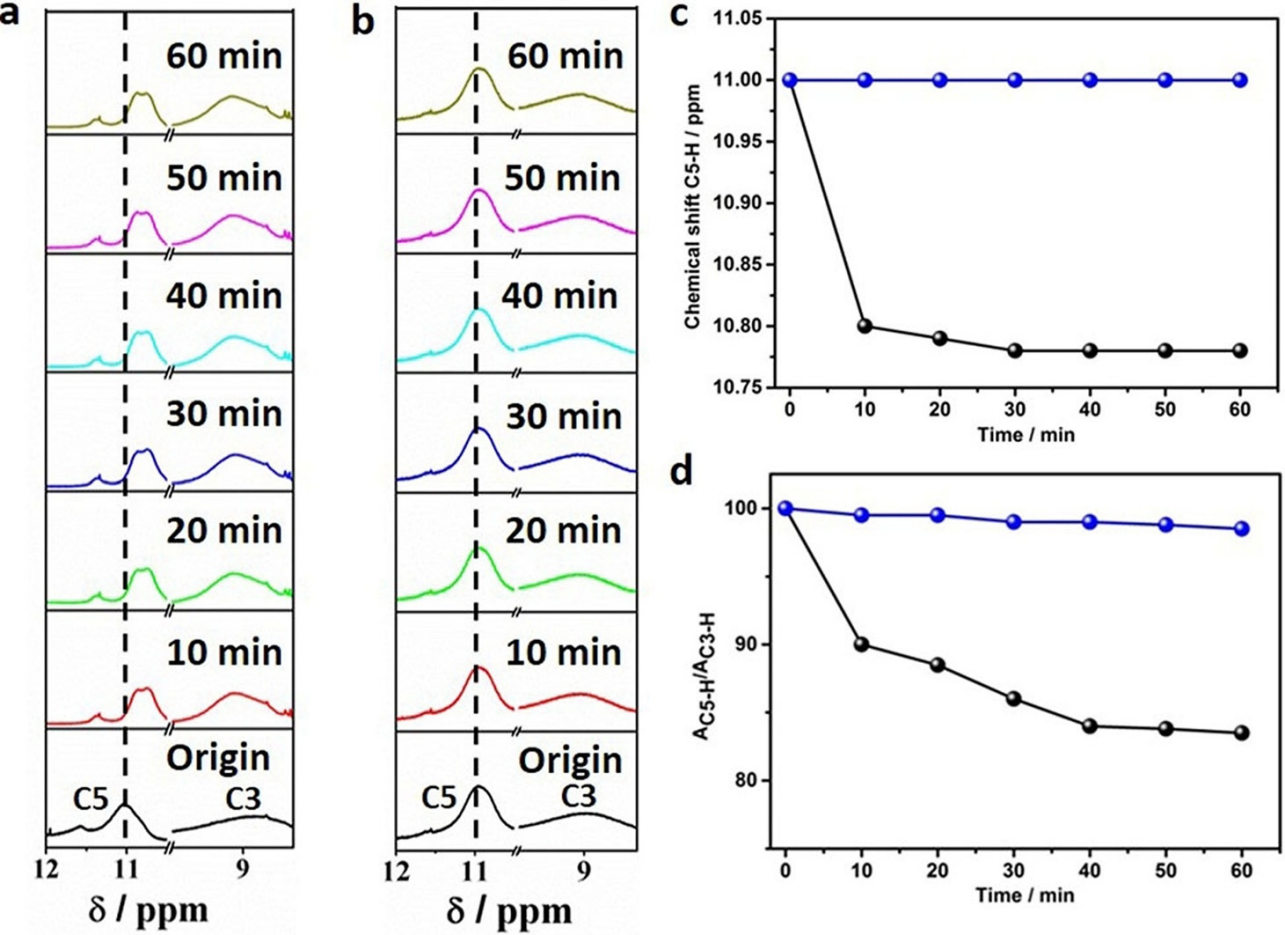
a) In situ ^1^H NMR spectra of C5 and C3 protons in Ptriaz during the CC3R synthesis in deuterated chloroform at room temperature. b) In situ ^1^H NMR spectra of C5 and C3 protons in Ptriaz in deuterated chloroform at room temperature. c) Chemical shift of C5 protons in Ptriaz against time. Blue line: without CC3R, black line: with CC3R crystallizing process. d) The integration of C5 and C3 proton peaks in Ptriaz. Blue line: without CC3R, black line: with CC3R crystallizing process.

To pinpoint the promoting role of Pitraz in crystallization, time‐dependent proton nuclear magnetic resonance (^1^H NMR) spectra of the reaction mixture for the synthesis of CC3R by Ptriaz in deuterated chloroform were collected in situ. It showed that the signal of C5‐proton in the triazolium ring at 11.00 ppm shifts to an upfield at 10.80 ppm within 10 min (Figure 3 a; Supporting Information, Figure S9), indicative of an added shielding effect due to the precursor‐Ptriaz interaction.^[19]^ In the next 50 min along the reaction time, the δ of C5 proton basically maintains at 10.80±0.03 ppm. By contrast, the C5 proton does not shift at all for 60 min after Ptriaz was added alone in deuterated chloroform (Figure 3 b; Supporting Information, S10). In fact, the upfield shift of the C5‐proton signal was also observed even when mixing Ptriaz only with the precursor either BTA or DACH separately (Supporting Information, Figures S11, S12). Such shift is particularly profound in the Ptriaz‐DACH mixture (from 11.00 to 10.87 ppm) after 10 min of mixing (Supporting Information, Figure S12), followed by a simultaneous down‐field shift of signal in DACH (Supporting Information, Figure S13). It is easily understood that as a potential electron donor, ‐NH_2_ can form hydrogen bonding (C−H⋅⋅⋅N) with the triazolium moiety, and a similar shift has been experimentally reported in an imidazolium‐electron donor system, and theoretical calculation indicated an increased length of C−H in the present of DACH (see computation section in the Supporting Information and Figure S32 therein).^[20]^ We further analyze the integral ratio of the C5 to C3 proton (as a reference peak without proton loss) in the triazolium ring during the imine bond formation reaction. A sharp loss of about 10 % of C5 proton could be clearly observed in the initial 10 min, after which only a gradual loss was seen. In comparison, no change was detected in pure Ptriaz system (Figure 3 d) in the same period, which suggests that the C5 proton participates in catalyzing the imine bond formation reaction. It is well known that the formation of an imine bond from an aldehyde and primary amine is a reversible reaction and generally can take place under acid catalysis.^[21]^ In our current system, the interaction between DACH and Ptriaz favorably impairs the strength of the C−H bond, facilitating ionization of the C5 proton and enhancing its acidity that better catalyzes the imine bond formation reaction.^[20c]^ Such hydrogen bonding activation of substance has also been observed in MOF synthesis in ionic liquid in which the hydrogen bonding (C−H⋅⋅⋅O) interaction between the C2 proton of imidazolium ring and the modulator (acetic acid) activated the linker exchange, and thus to accelerate the coordination crystallization.^[20a]^ The results of ^1^H NMR spectra for the CC3R synthesis in the presence of other triazolium bearing PILs (Supporting Information, Figure S14) are similar to that of Ptriaz, implying that CC3R undergoes the same crystallization procedure in the presence poly(1,2,4‐triazolium)s. Similar change in the ^1^H NMR spectra could be found in other COF‐PIL and macrocycle‐PIL crystallization systems (Supporting Information, Figures S15–S17), that is, a general effect of poly(1,2,4‐triazolium)s.

**Figure 4:**
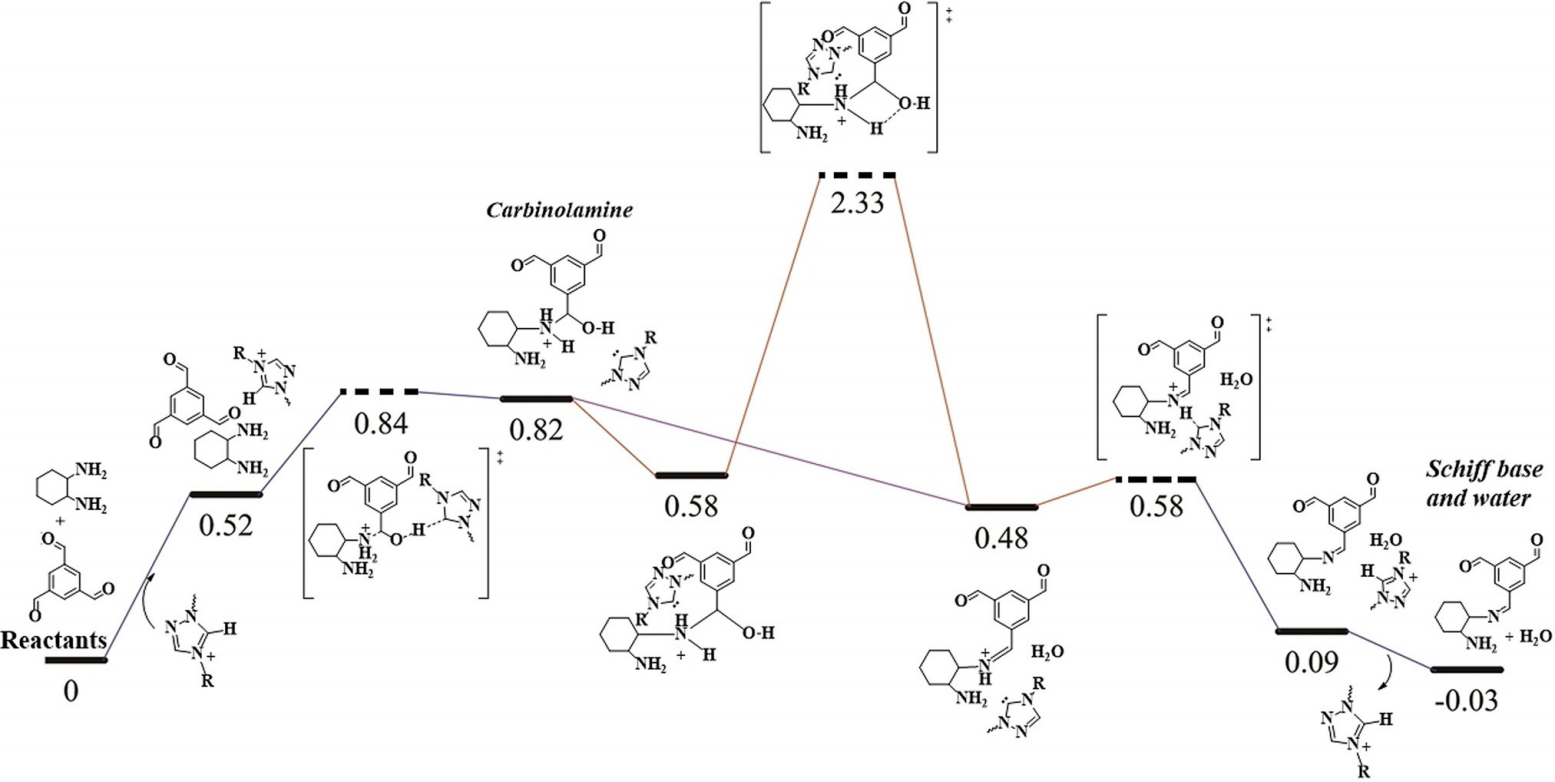
Energy profiles of the reaction path with catalyst (the 1,2,4‐triazolium monomer was used here for calculation). The horizontal dashed line represents the transition states, and the relative energies (Δ*E*) are noted above the line.

To further elucidate the underlying mechanism of imine bond formation in the presence of Ptriaz catalyst, density functional theory (DFT) calculations on the reaction between DACH and BTA were performed, and the details were shown in Figure 4 and computation section in Supporting Information. Influenced by the Triaz unit, the amine as a nucleophile first attacks the aldehyde to give an unstable carbinolamine intermediate (step I). This step has an energy barrier (*E*_a_) of 0.32 eV and is endothermic (Δ*E*=0.30 eV). The further dehydration of the carbinolamine forms a stable imine species, which is proton‐catalyzed (the purple line in Figure 4). This process is energy barrierless, and exothermic (Δ*E*=−0.34 eV), suggesting that the rate‐limiting step of the overall reaction is the formation of the C−N bond (step II).^[22]^

The chemical structure effect of PILs on the crystallization rate was analyzed by carrying out the same synthetic procedure in the presence of imidazolium‐ and pyridinium‐based PILs, for example, poly(4‐hexyl‐1‐vinylimidazolium iodide) (denoted as PIL‐imidaz), and poly(4‐hexyl‐1‐vinylpyridinium iodide) (denoted as PIL‐py). The crystallization rate follows an order of Ptriaz (5 min) > PIL‐imidaz (30 min) > PIL‐py (8 h), in which the time indicates how long the CC3R crystals reach an average size of about 2 μm (Supporting Information, Figure S18). The afore‐mentioned ^1^H‐NMR experiment on kinetic hydrogen–deuterium exchange in D_2_O supports that the imidazolium C2‐proton is slower to be exchanged than the 1,2,4‐triazolium C‐5 proton, that is, the latter is far more active.^[13b]^ Such statement is consistent with the observation of the slower up‐field shift of the imidazolium C2 proton in the PIL‐imidaz‐CC3R crystallization system (Supporting Information, Figure S19). For the CC3R‐PIL‐py crystallization system, there is no active proton in the pyridinium ring to catalyze the imine bond formation reaction, which corresponds to the slowest crystallization process.

The effect of the polymeric nature of PILs on accelerating the crystallization process was investigated in a control experiment using its monomer unit 4‐hexyl‐1‐vinyl‐1,2,4‐triazolium bis(trifluoromethane sulfonyl)imide) at an identical monomer/repeating unit concentration. It took 20 min to reach a crystal size of about 2 μm, which is 3 times longer than its polymer Ptriaz. The signal shift in the ^1^H NMR spectra and loss of C5 proton upon reaction time were spotted and supported the role of C5 proton as catalyst for the imine bond reaction (Supporting Information, Figure S20). Taking consideration that the same amount of triazolium units was used, the macromolecular architecture of PILs does improve their catalytic effect, very possibly because of a stronger solvation effect than that of monomer, an effect that is well known in polymer physical chemistry.^[23]^ The stronger solvation capability of polymers can effectively compete for solvent molecules with the solute which, upon losing the solvent, starts to crystallize faster than that of monomer. Note that such effect is reminiscent of salting out causing precipitation and/or crystallization^[24]^ of various solutes in purification industry.

**Figure 5:**
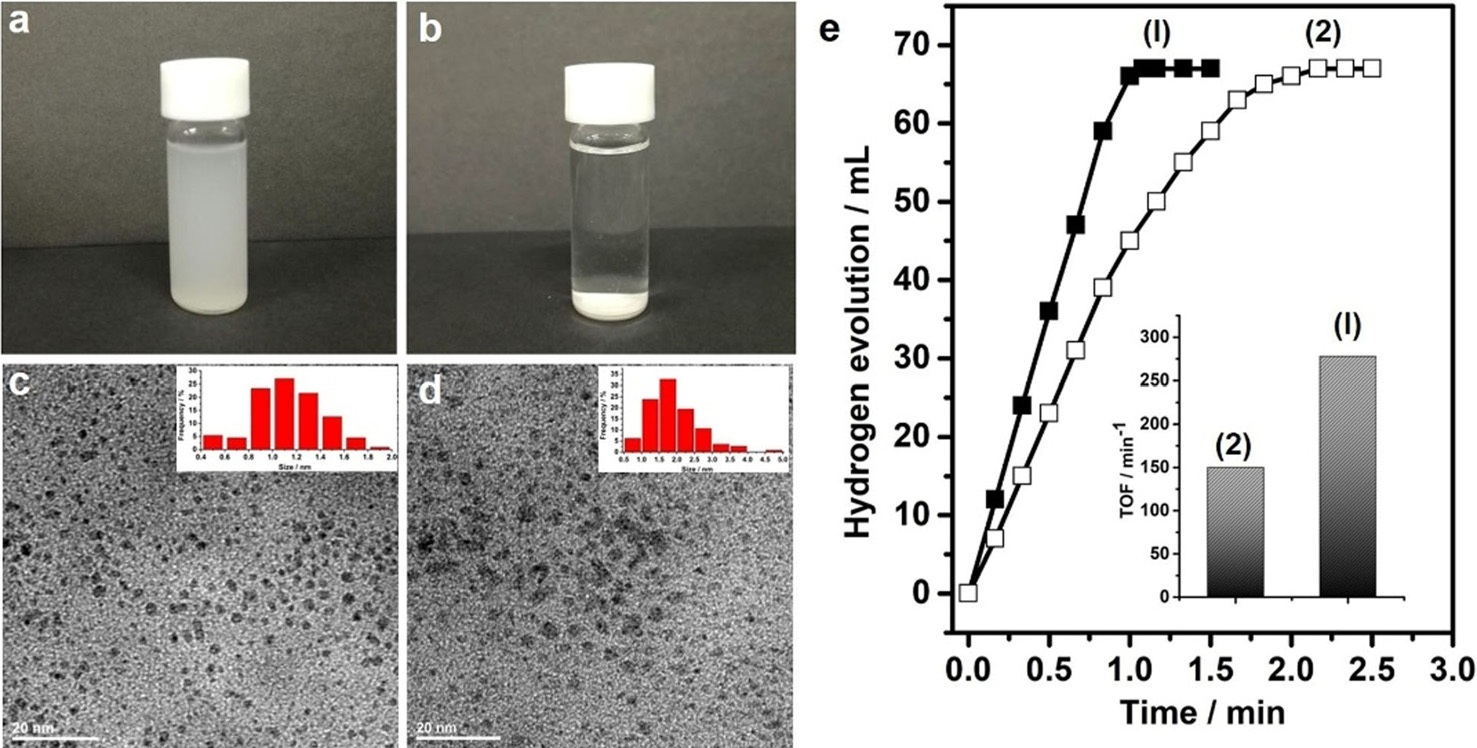
a),b) Comparison of dispersibility of CC3R synthesized with Ptriaz (a) and without Ptriaz (b) additive in methanol. c),d) The corresponding TEM images of Rh cluster stabilized by two above‐mentioned kinds of CC3R. e) Time‐course plots of H_2_ generation for the methanolysis of ammonia borane (AB) over the Rh/CC3R‐Ptriaz (solid dark, 1) and Rh/CC3R (blank dark, 2) at 298 K (Rh/AB=0.01). Inset: corresponding TOF values.

The PIL is well‐known as surface‐active polymers toward different substances through Coulombic interaction, cation–π interaction, van der Waals’ force, or other charge‐polarized interactions.[^12d^, ^25^] When CC3R crystals are grown in the presence of PIL, ca. 2 wt % of Ptriaz was measured to be immobilized on the crystal surface as indicated by elemental analysis. X‐ray photoelectron spectroscopy (XPS) analysis identifies the interaction between CC3R and Ptriaz. The 3d signal of I, which originates only from Ptriaz, showing a detectable 1.0 eV shift from 630 eV in native Ptriaz to a lower binding energy position at 629 eV in the CC3R‐Ptriaz product (Supporting Information, Figure S21), suggesting that PIL chains are coupled to the CC3R surface. The crystalline surface associated with PIL chains provides a routine for dispersion of crystalline materials in liquid; here in methanol, a good solvent for Ptriaz, was employed for test. As shown in Figure 5 a, 5 mg of CC3R synthesized in the presence of Ptriaz can be fairly dispersed in 4 mL of methanol after a gentle sonication treatment, while the similarly synthesized CC3R of the same crystal size without Ptriaz precipitated in methanol within 1 min (Figure 5 b) even after long‐time sonication.

It is well‐known that the crystalline materials as heterogeneous catalysts have a strong tendency to aggregate in solution, which inevitably cause retarded mass transfer, reduced interfacial area and consecutively hampered catalytic capability in liquid‐phase catalysis.^[26]^ The well‐dispersed system provides us a routine to solve such problem once in combination with the metal nanoparticles/clusters for liquid‐phase catalytic reaction. Here, the CC3R‐immobilized Rh cluster for AB methanolysis reaction was employed as a model test. The Rh/CC3R‐Ptriaz (particle size: 1.1±0.2 nm) shows a turnover frequency (TOF) of 278 min^−1^ (Figure 5 c,e), which is comparable to the highest activity among the cage immobilized catalysts for AB methanolysis reaction (304.4 min^−1^ for Ru NPs@PCC‐2 catalyst).^[27]^ Such activity is 1.9 times higher than that of the CC3R without Ptriaz (TOF: 150 min^−1^, particle size: 1.9±0.5 nm) (Figure 5 d,e), or 21 times higher than support‐free Rh catalyst with aggregated particles (TOF: 13 min^−1^) (Supporting Information, Figures S22, S23). Since the cage and PIL are inert to the reaction (Supporting Information, Figures S24, S25), such enhanced activity could be reasonably assigned to the enhanced dispersibility in solution as well as the smaller size of Rh clusters, which would facilitate the mass transfer between the Rh and reactants.

## Conclusion

Kinetically promoted room‐temperature crystallization of several imine‐linked open organics by 1,2,4‐triazolium PIL as a simple additive is reported. The active C5 proton in 1,2,4‐triazolium units serving as acid together with the strong solvation power of polyelectrolytes can jointly catalyze the reaction and precipitate out the formed crystalline products without deteriorating their crystallinity. This previously undiscovered mechanism is important and will inspire porous materials community to expedite the research progress of open organic materials.

## Conflict of interest

The authors declare no conflict of interest.

## Supporting information

As a service to our authors and readers, this journal provides supporting information supplied by the authors. Such materials are peer reviewed and may be re‐organized for online delivery, but are not copy‐edited or typeset. Technical support issues arising from supporting information (other than missing files) should be addressed to the authors.

Supplementary
