# Supplementary material for: Accelerating Crystallization of Open Organic Materials by Poly(ionic liquid)s
Source: Angew Chem Int Ed Engl. 2020 Oct 4;59(49):22109–16. doi: 10.1002/anie.202008415 (PMC7756458; doi:10.1002/anie.202008415)
Supplement: Supplementary file 1 — Supplementary [file ANIE-59-22109-s001.pdf]

Angewandte Chemie
Eine Zeitschrift der Gesellschaft Deutscher Chemiker

Supporting Information

Accelerating Crystallization of Open Organic Materials by Poly(ionic liquid)s

Su-Yun Zhang+, Han Miao+, He-min Zhang+, Jun-Hao Zhou, Qiang Zhuang, Yu-Jia Zeng, Zhiming Gao, Jiayin Yuan, and Jian-Ke Sun**

anie_202008415_sm_miscellaneous_information.pdf

Experimental Procedures

1. Chemicals and Instrumentation

All chemicals were from commercial sources and used without further purification.

Transmission electron microscopy (TEM) was performed on a JEOL 2010FS transmission electron microscope operated at 120 kV. Powder X-ray diffraction (PXRD) was carried out on an X-ray diffractometer of Rigaku, Ultima IV. X-ray photoelectron spectroscopy (XPS) studies were performed on a ThermoFisher ESCALAB250 X-ray photoelectron spectrometer (powered at 150 W) using Al K_α radiation (λ = 8.357 Å). To compensate for surface charging effects, all XPS spectra were referenced to the C 1s neutral carbon peak at 284.6 eV. ^{1}H nuclear magnetic resonance (^{1}H-NMR) measurements were carried out at room temperature on a Bruker DPX-400 spectrometer in different deuterated solvents. The N_2 sorption isotherms were measured on automatic volumetric adsorption equipment (Belsorp-mini II).

2. Experimental Section

2.1 Synthesis of poly(ionic liquid)s

The preparation methods of 4-hexyl-1-vinyl-1,2,4-triazolium iodide and poly(4-hexyl-1-vinyl-1,2,4-triazolium iodide) (denoted as "Ptriaz") were described in the literature (J. K. Sun, Z. Kochovski, W. Y. Zhang, H. Kirmse, Y. Lu, M. Antonietti, J. Yuan, *J. Am. Chem. Soc.*, 2017, 139, 8971–8976.).

The preparation methods of 4-octyl-1-vinyl-1,2,4-triazolium iodide and poly(4-octyl-1-vinyl-1,2,4-triazolium iodide) were described as follows:

Synthesis of 4-octyl-1-vinyl-1,2,4-triazolium iodide: A mixture of 1-vinyl-1,2,4-triazole 1 (5 mL, 5.5g, 57.83 mmol) and a 1.2 equivalent amount of 1-Iodooctane were added into a 100 mL round flask, accompanied with 2,6-di-tert-butyl-4-methylphenol (50 mg, 0.227 mol) as the stabilizer. After heated at 50 °C overnight, crude products were precipitated in diethyl ether and washed with the same solvent for three times. Pale yellow powders were obtained after purification.

Sythesis of poly(4-octyl-1-vinyl-1,2,4-triazolium iodide): A mixture of 4-octyl-1-vinyl-1,2,4-triazolium iodide monomer with AIBN (1.5 mol%) as initiator was added to anhydrous DMF (concentration: ~1 g monomer in 10 mL solvent) inside a 100 mL round-bottom schlenk flask. The flask was treated with three freeze-pump-thaw cycles and finally purged with argon. The reaction was stirred at 70 °C for 24 h under argon atmosphere. Yellow powders were obtained after dialysis against water and a vacuum drying process.

The preparation methods of 4-cyanomethyl-1-vinyl-1,2,4-triazolium bis(trifluoromethane sulfonyl)imide and poly(4-cyanomethyl-1-vinyl-1,2,4-triazolium bis(trifluoromethane sulfonyl)imide) were described in the literature (J. K. Sun, W. Zhang, R. Guterman, H. J. Lin, J. Yuan, *Nat. Commun.*, 2018, 9, 1717).

The preparation methods of 4-hexyl-1-vinyl-imidazolium iodide and poly(4-hexyl-1-vinyl-imidazolium iodide) (simplified as "PIL-imidaz") were described in the literature (J. C. Salamone, S. C. Israel, P. Taylor, B. Snider, *Polymer*, 1973, 14, 639-644.)

The preparation of poly(1-hexyl-4-vinyl-pyridinium iodide) (denoted as "PIL-py") is as follows: A mixture of commercially purchased poly(4-vinylpyridine) (3 g, Mw = 1.6 $\times 10^5$ g/mol) and a 1.5 equivalent amount of 1-Iodohexane in 50 mL of DMF was added into a 150 mL round flask. After heating at 80 °C for 10 h, dark brown powders were obtained upon precipitation in THF, then it was washed with the same solvent for three times. The chemical structure was confirmed by ^{1}H NMR spectrum in Figure S26.

2.2 Synthesis of open organics

Synthesis of CC3R with Ptriaz additive: For the typical synthesis of CC3R, 0.35 mL of chloroform solution containing 1,3,5-triformylbenzene (8 mg, 0.05 mmol) and Ptriaz (25 mg, 0.08 mmol) was added by a chloroform solution (0.35 mL) of (R,R)-1,2-diaminocyclohexane (8 mg, 0.07 mmol). The vessel of the mixture solution was capped and left to stand for a certain time to give out the single crystals. The precipitate crystals were collected and dried under vacuum at 80 °C for 24 h.

Synthesis of CC3R without Ptriaz additive: The synthetic procedure used above to prepare CC3R-Ptriaz was followed without Ptriaz additive.

Synthesis of CC3R with PIL-imidaz additive: The synthetic procedure used above to prepare CC3R-Ptriaz was followed by using PIL-imidaz containing chloroform instead of Ptriaz.

Synthesis of CC3R with PIL-py additive: The synthetic procedure used above to prepare CC3R-Ptriaz was followed by using PIL-py containing chloroform instead of Ptriaz.

Synthesis of CC3R with triaz monomer additive: The synthetic procedure used above to prepare CC3R-Ptriaz was followed by using triaz monomer containing chloroform instead of Ptriaz.

Synthesis of BTA-PED COF with poly(4-cyanomethyl-1-vinyl-1,2,4-triazolium bis(trifluoromethane sulfonyl)imide) additive: For the typical synthesis of BTA-PED COF, 0.4 mL of dioxane solution containing 1,3,5-triformylbenzene (10 mg, 0.06 mmol) and 1,4-phenylenediamine (PED) (10 mg, 0.09 mmol), and then 0.4 mL of dmso solution containing poly(4-cyanomethyl-1-vinyl-1,2,4-triazolium bis(trifluoromethane sulfonyl)imide) (30 mg, 0.07 mmol) was added. The vessel of the mixture solution was capped and left to stand for a certain time to give out the BTA-PED COF precipitate. The precipitate crystals were collected by centrifugation and dried under vacuum at 80 °C for 24 h.

Synthesis of BTA-PED COF without poly(4-cyanomethyl-1-vinyl-1,2,4-triazolium bis(trifluoromethane sulfonyl)imide) additive: The synthetic procedure used above to prepare BTA-PED COF without any additive.

Synthesis of TPA-DACH macrocycle with poly(4-octyl-1-vinyl-1,2,4-triazolium iodide) additive: For the typical synthesis of TPA-DACH macrocycle, 0.3 mL of chloroform solution containing terephthalaldehyde (TPA) (13.4 mg, 0.1 mmol) and poly(4-octyl-1-vinyl-1,2,4-triazolium iodide) (25 mg,

0.08 mmol) was added by a chloroform solution (0.3 mL) of (R,R)-1,2-diaminocyclohexane (11.4 mg, 0.1 mmol). The vessel of the mixture solution was capped and left to stand for a certain time to give out the single crystals. The precipitate crystals were collected by centrifugation and dried under vacuum at 80 °C for 24 h.

Synthesis of TPA-DACH macrocycle without poly(4-octyl-1-vinyl-1,2,4-triazolium iodide) additive: The synthetic procedure used above to prepare TPA-DACH macrocycle without any additive.

Synthesis of IPA-DACH macrocycle with Ptriaz additive: For the typical synthesis of IPA-DACH macrocycle, 0.3 mL of chloroform solution containing isophthalaldehyde (IPA) (13.4 mg, 0.1 mmol) and Ptriaz (25 mg, 0.08 mmol) was added by a chloroform solution (0.3 mL) of (R,R)-1,2-diaminocyclohexane (11.4 mg, 0.1 mmol). The vessel of the mixture solution was capped and left to stand for a certain time to give out the single crystals. The precipitate crystals were collected by centrifugation and dried under vacuum at 80 °C for 24 h.

Synthesis of IPA-DACH macrocycle without Ptriaz additive: The synthetic procedure used above to prepare IPA-DACH macrocycle without any additive.

2.3 Synthesis of catalyst for AB methanolysis reaction

Synthesis of Rh/CC3R-Ptriaz catalyst. In a typical synthesis, 4 mL of methanol containing 5 mg of CC3R (with Ptriaz additives, denoted as CC3R-Ptriaz) was subsequently added to 0.5 mL of methanol containing $Rh(OAc)_3$ (0.01 mol Rh in content). The resultant mixture solution was further homogenized after aging for 20 min. Then, 0.5 mL of methanol solution containing 5 mg of $NaBH_4$ was immediately added into the above solution with vigorous shaking, resulting in a well dispersion of Rh/CC3R-Ptriaz.

Synthesis of Rh/CC3R catalyst. The synthetic procedure used above to prepare Rh/CC3R-Ptriaz catalyst was followed by using 4 mL of methanol with as-synthesized CC3R as stabilizer.

Synthesis of Rh-SP-Free catalyst. The synthetic procedure used above to prepare Rh/CC3R-Ptriaz catalyst was followed by using 4 mL of methanol without any stabilizer.

2.4 Catalytic activity characterization

Procedure for the methanolysis of AB by Rh/CC3R-Ptriaz catalyst: The reaction apparatus for measuring the hydrogen evolution from the methanolysis of AB is as follows. In general, the as-synthesized Rh/CC3R-Ptriaz catalyst was placed in a two-necked round-bottomed flask (30 mL), which was placed in a water bath under ambient atmosphere. A gas burette filled with water was connected to the reaction flask to measure the volume of hydrogen. The reaction started when AB (30.8 mg) in 0.8 mL of methanol was added into the flask. The volume of the evolved hydrogen gas was monitored by recording the displacement of water in the gas burette. The reaction was completed when there was no more gas generation. The methanolysis of AB can be expressed as follows:

$$NH_3BH_3 + 4CH_3OH \rightarrow NH_4B(CH_3O)_4 + 3H_2 \qquad (1)$$

Procedures for the methanolysis of AB by Rh-CC3R catalyst and Rh-SP-Free catalysts: The procedures for the methanolysis of AB were similar to that of Rh/CC3R-Ptriaz catalyst except different catalysts were used.

3. Results and Discussion

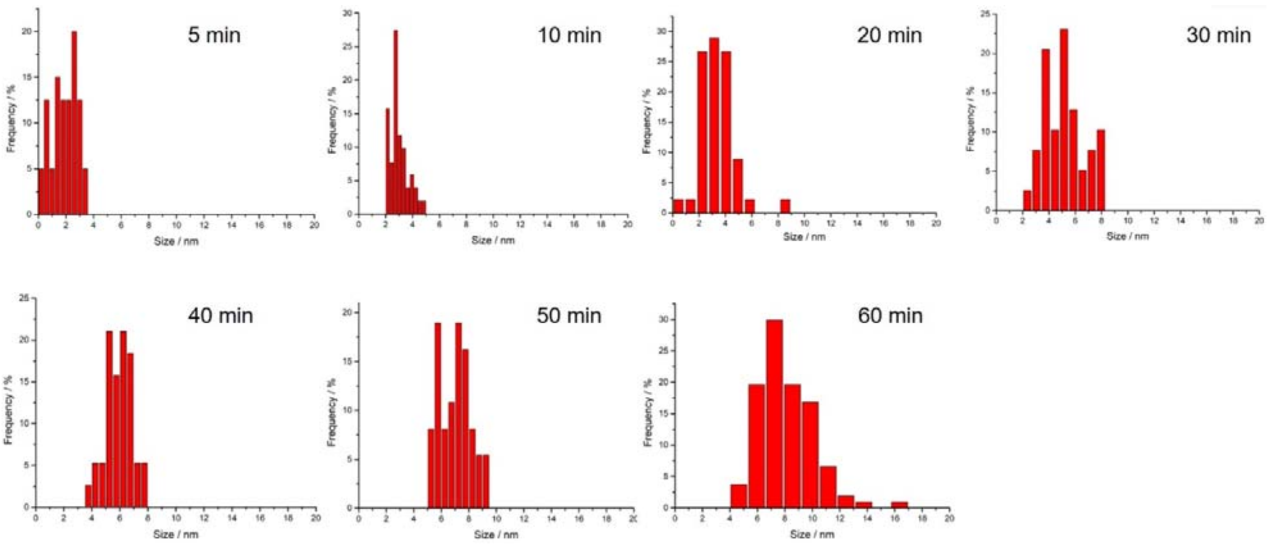

Figure S1. Time dependent size-distribution of CC3R Crystals in the presence of Ptriaz.

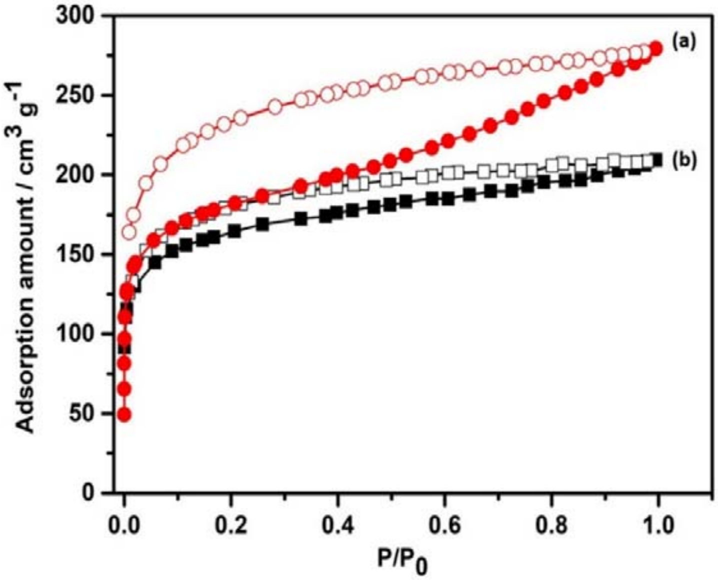

Figure S2. The N_2 sorption isotherms of (a) CC3R-Ptriaz and (b) CC3R (without acid catalyst) at 77 K. Filled and open symbols represent adsorption and desorption branches, respectively.

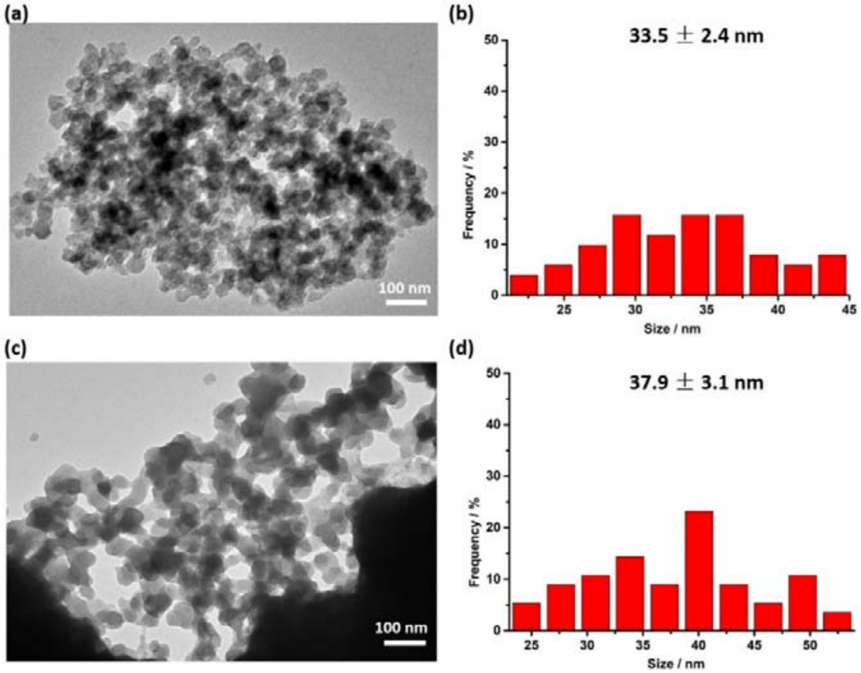

Figure S3. Images of crystallites and corresponding size distributions of BTA-PED COF with (a,b) and without (c,d) poly(4-cyanomethyl-1-vinyl-1,2,4-triazolium bis(trifluoromethane sulfonyl)imide) after reaction for 5 and 300 min, respectively.

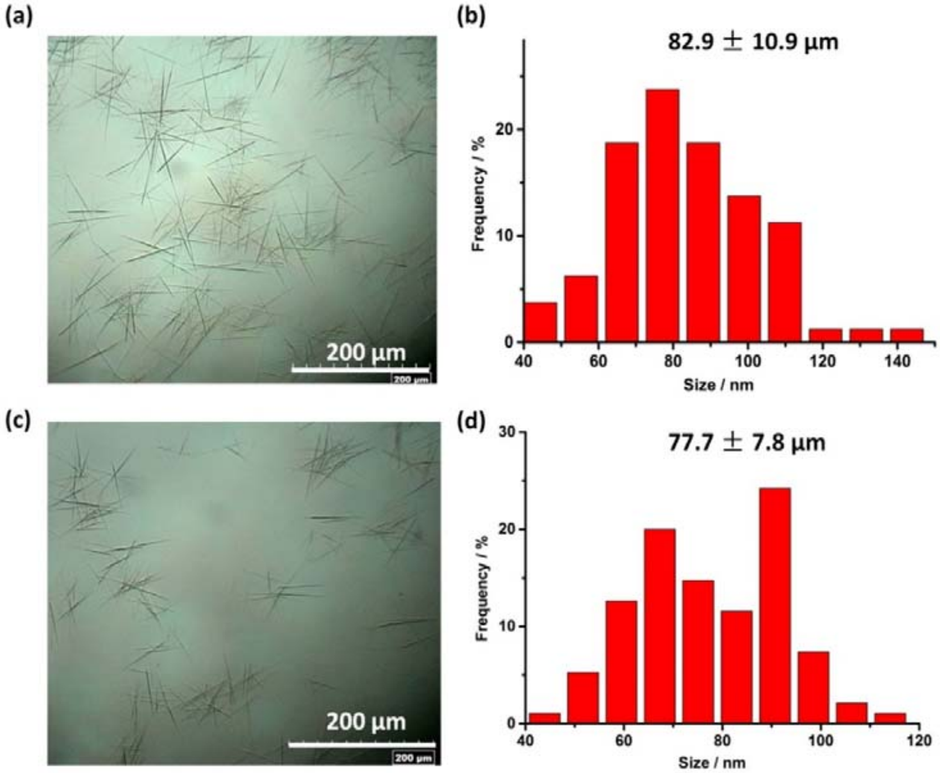

Figure S4. Images of crystals and corresponding size distributions of TPA-DACH macrocycle single crystals with (a,b) and without (c,d) poly(4-octyl-1-vinyl-1,2,4-triazolium iodide) after reaction for 30 and 300 min, respectively.

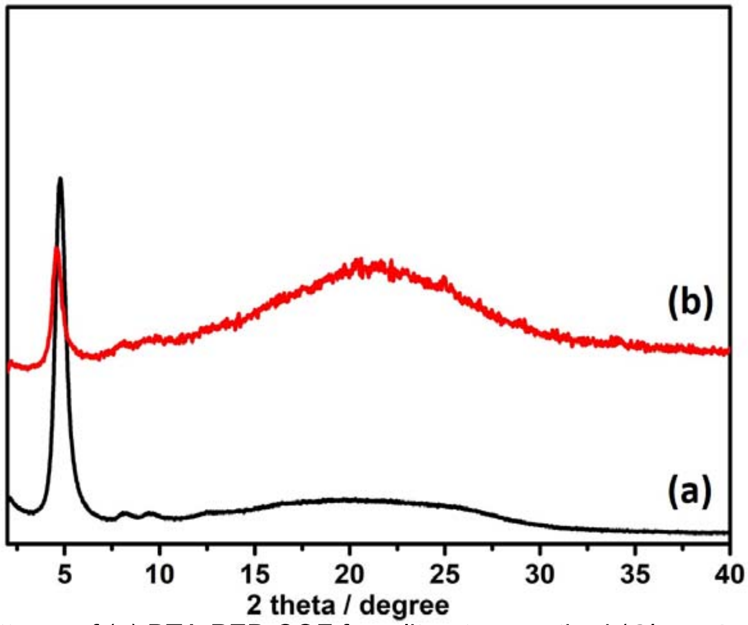

Figure S5. PXRD patterns of (a) BTA-PED COF from literature method (*Chem. Lett.* 2015, 44, 1488–1490), (b) as-synthesized one in the presence of PIL additive.

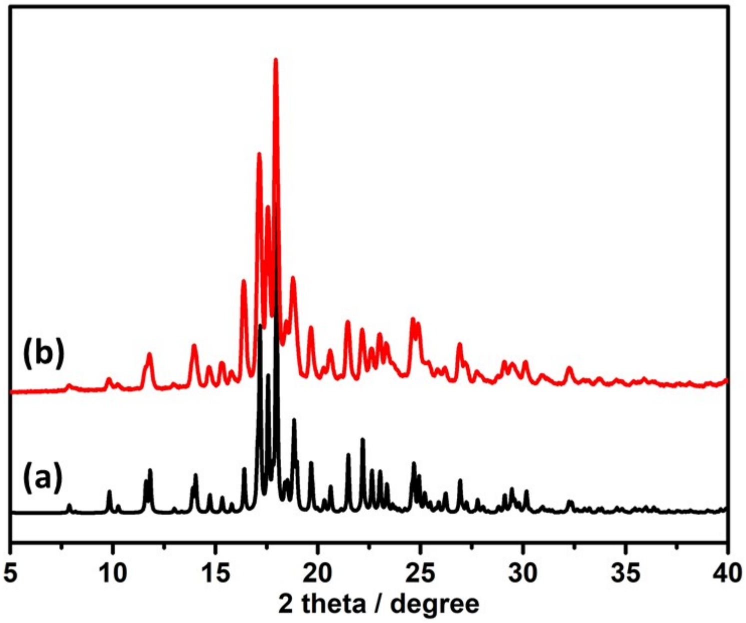

Figure S6. PXRD patterns of (a) simulated TPA-DACH macrocycle single crystal re-crystallized from ethyl acetate from literature (*J. Org. Chem.* 2000, 65, 5768-5773), (b) as-synthesized one in the presence of PIL additive, which was further re-crystallized from ethyl acetate.

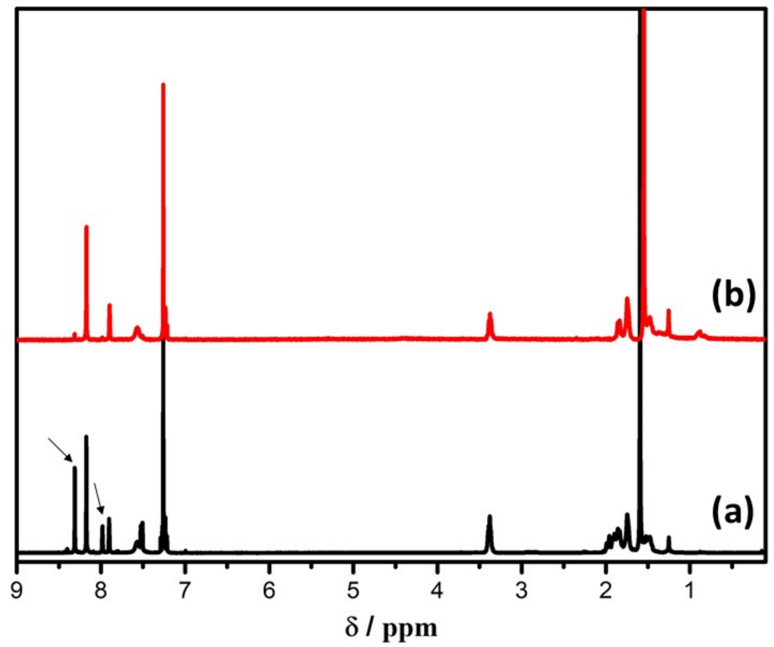

Figure S7. The ^{1}H NMR spectra of IPA-DACH macrocycle crystals synthesized in PIL-free condition without further purification (a), the [2+2] cyclocondensation byproduct with signal at 8.3 and 7.9 ppm (*Chem. Commun.* 2010, 46, 4315-4317) could be detected with estimated amount of ~ 45 % in the final crystals, while such thermodynamic byproduct could be effectively suppressed in the presence of PIL catalyst (less than 4 %) and without further purification (b).

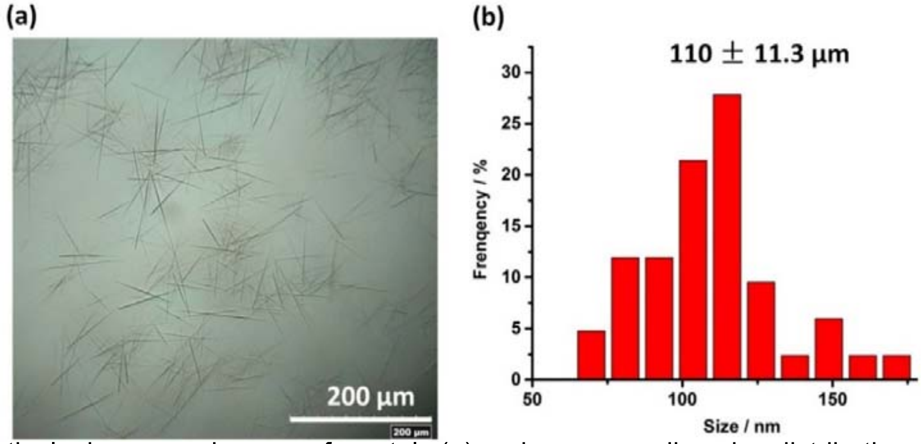

Figure S8. Optical microscopy images of crystals (a) and corresponding size distributions (b) of IPA-DACH macrocycle obtained in the presence of PIL.

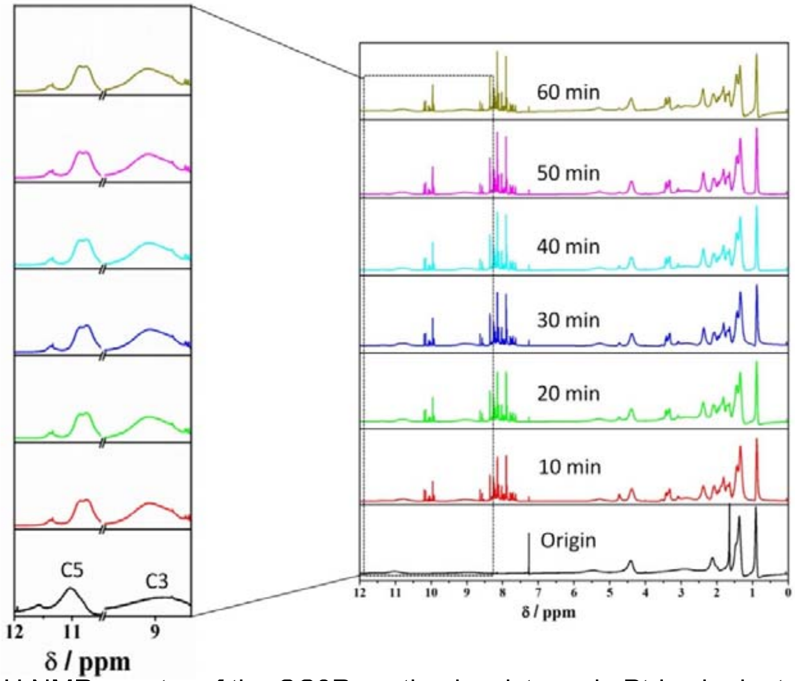

Figure S9. In situ ^{1}H NMR spectra of the CC3R synthesis mixtures in Ptriaz in deuterated chloroform at room temperature. The corresponding C5- and C3-protons in Ptriaz during the CC3R synthesis with higher magnification was highlighted in left column.

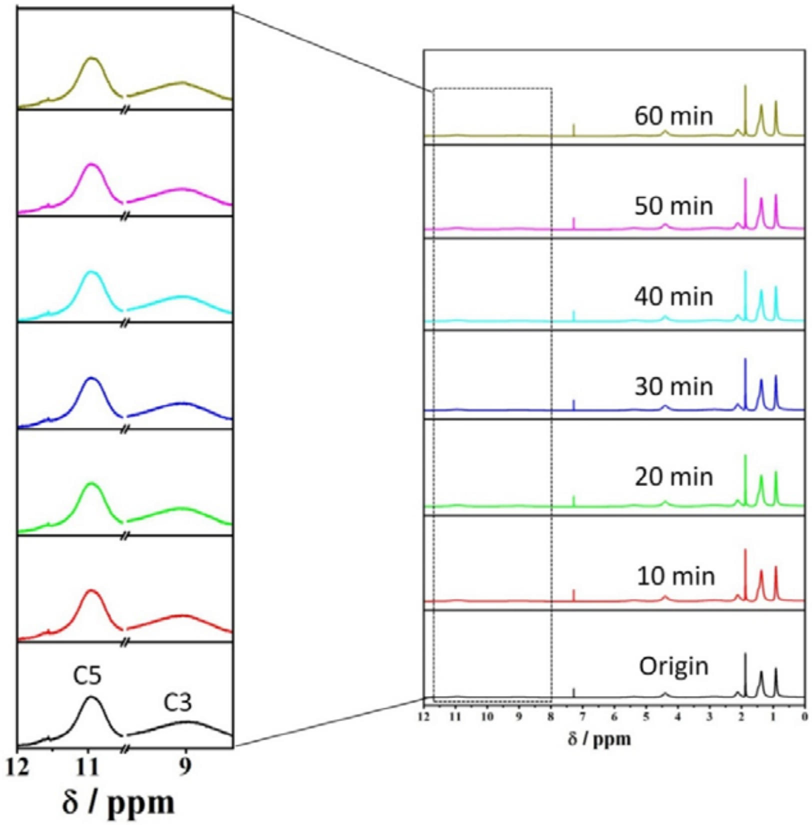

Figure S10. In situ ^{1}H NMR spectra of pure Ptriaz in deuterated chloroform at room temperature. The corresponding C5- and C3-protons in Ptriaz with higher magnification was highlighted in left column.

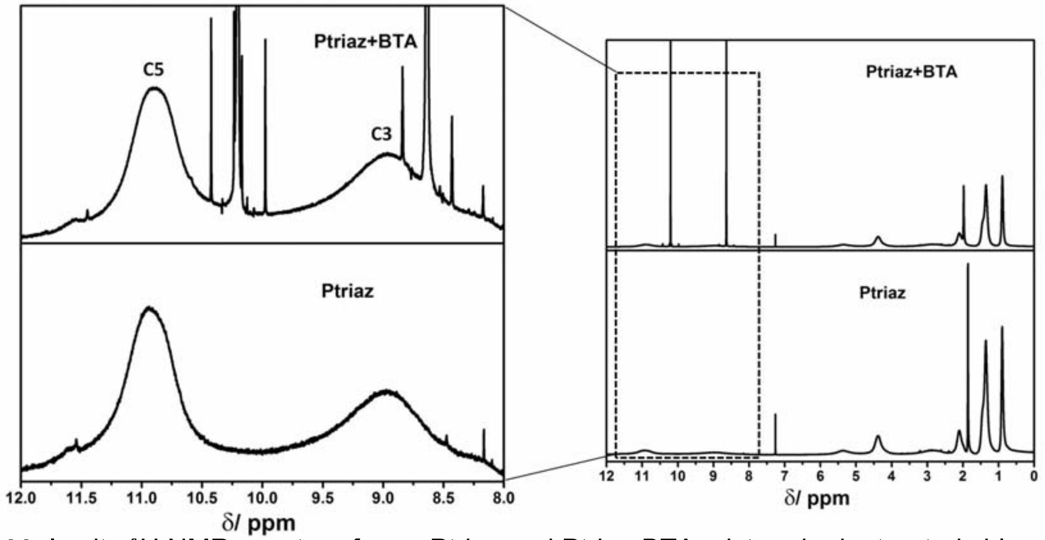

Figure S11. In situ ^{1}H NMR spectra of pure Ptriaz and Ptriaz-BTA mixture in deuterated chloroform at room temperature. The corresponding C5- and C3-protons with higher magnification was highlighted in left column.

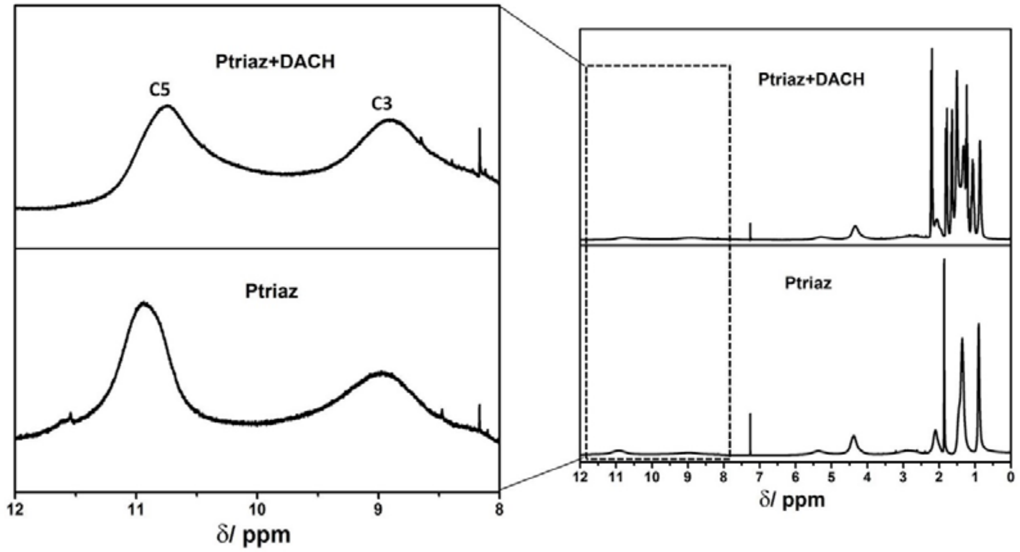

Figure S12. In situ ^{1}H NMR spectra of pure Ptriaz and Ptriaz-DACH mixture in deuterated chloroform at room temperature. The corresponding C5- and C3-protons with higher magnification was highlighted in left column.

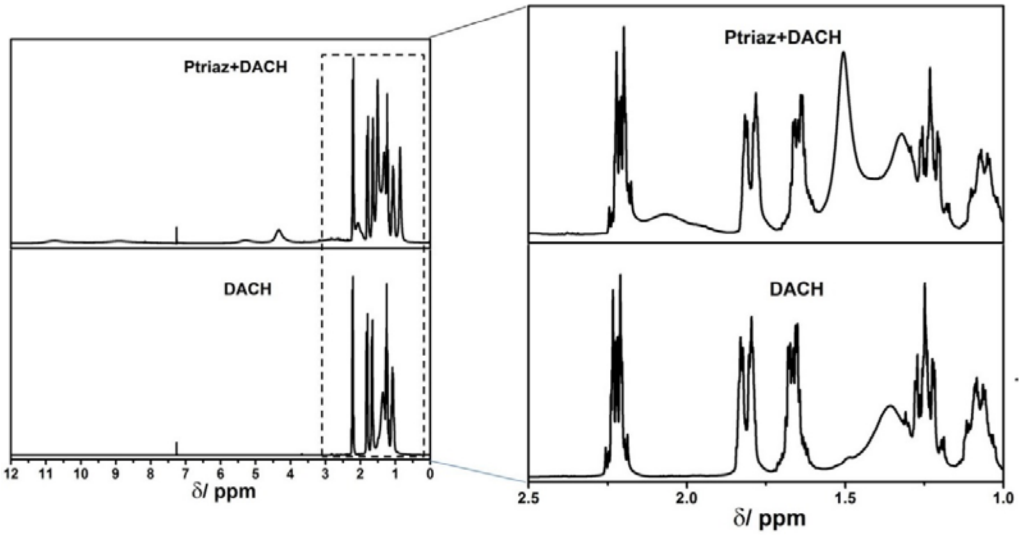

Figure S13. In situ ^{1}H NMR spectra of pure DACH and Ptriaz-DACH mixture in deuterated chloroform at room temperature. The higher magnification was highlighted in right column.

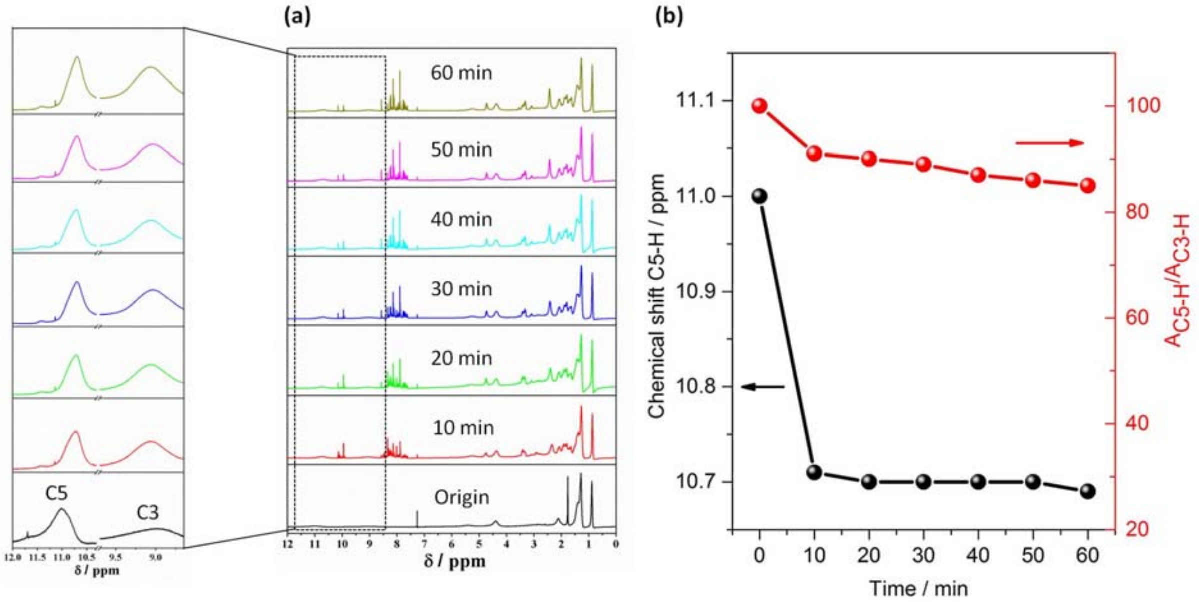

Figure S14. a, In situ ^{1}H NMR spectra of the CC3R synthesis mixtures in poly(4-octyl-1-vinyl-1,2,4-triazolium iodide) in deuterated chloroform at room temperature. The corresponding C5- and C3-protons in poly(4-octyl-1-vinyl-1,2,4-triazolium iodide) during the CC3R synthesis with higher magnification was highlighted in left column. b, Chemical shift (dark line) and integration of C5- proton peaks (red line) in poly(4-octyl-1-vinyl-1,2,4-triazolium iodide) against time.

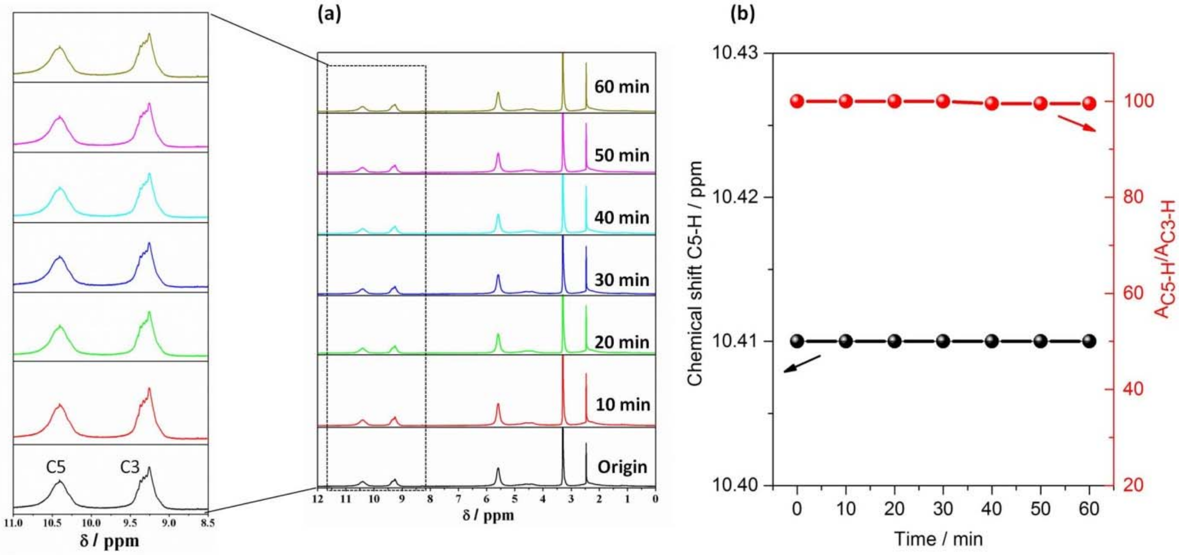

Figure S15. a, In situ ^{1}H NMR spectra of pure poly(4-cyanomethyl-1-vinyl-1,2,4-triazolium bis(trifluoromethane sulfonyl)imide) in deuterated DMSO at room temperature. The corresponding C5- and C3-protons in poly(4-cyanomethyl-1-vinyl-1,2,4-triazolium bis(trifluoromethane sulfonyl)imide) with higher magnification was highlighted in left column. b, Chemical shift (dark line) and integration of C5-proton peaks (red line) in poly(4-cyanomethyl-1-vinyl-1,2,4-triazolium bis(trifluoromethane sulfonyl)imide) against time.

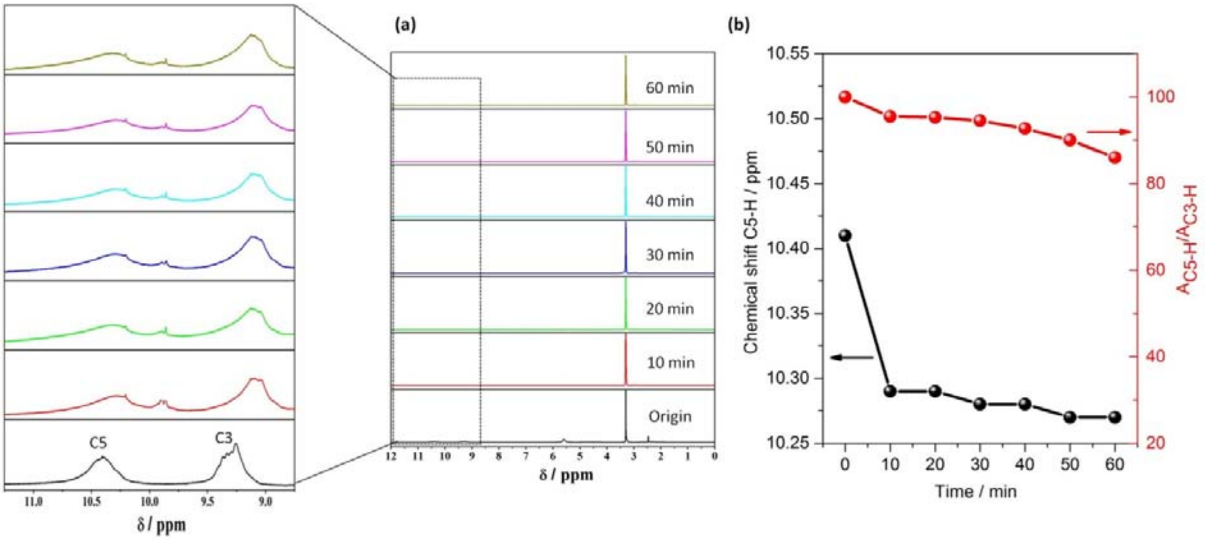

Figure S16. a, In situ ^{1}H NMR spectra of the BTA-PED COF synthesis mixtures in poly(4-cyanomethyl-1-vinyl-1,2,4-triazolium bis(trifluoromethane sulfonyl)imide) in deuterated DMSO at room temperature. The corresponding C5- and C3-protons in poly(4-cyanomethyl-1-vinyl-1,2,4-triazolium bis(trifluoromethane sulfonyl)imide) during the BTA-PED COF synthesis with higher magnification was highlighted in left column. b, Chemical shift (dark line) and integration of C5- proton peaks (red line) in poly(4-cyanomethyl-1-vinyl-1,2,4-triazolium bis(trifluoromethane sulfonyl)imide) against time.

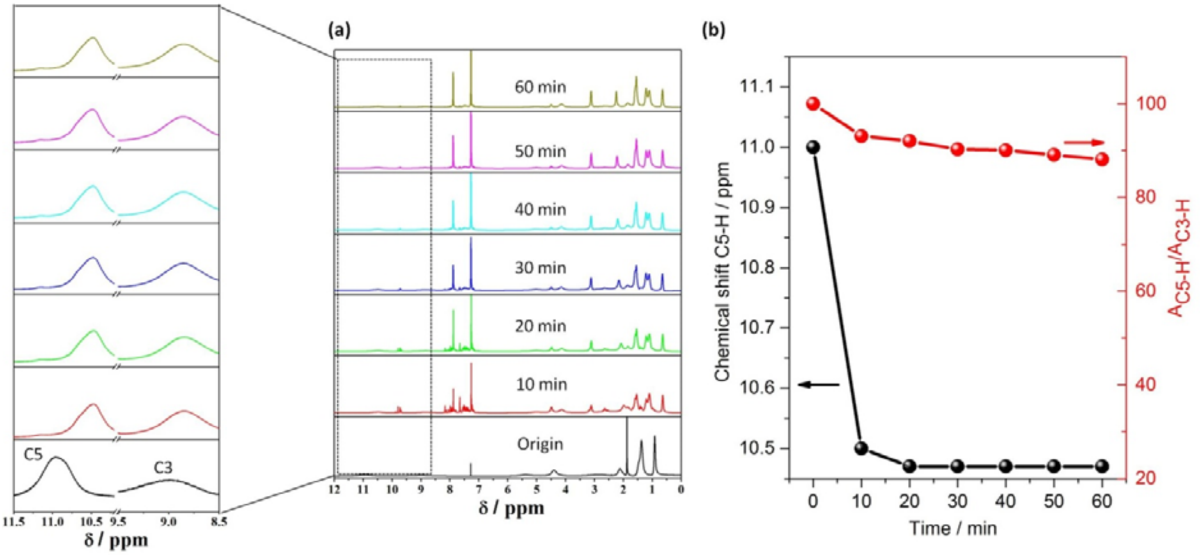

Figure S17. a, In situ ^{1}H NMR spectra of the TPA-DACH macrocycle synthesis mixtures in poly(4-octyl-1-vinyl-1,2,4-triazolium iodide) in deuterated chloroform at room temperature. The corresponding C5- and C3-protons in poly(4-octyl-1-vinyl-1,2,4-triazolium iodide) during the TPA-DACH macrocycle synthesis with higher magnification was highlighted in left column. b, Chemical shift (dark line) and integration of C5- proton peaks (red line) in poly(4-octyl-1-vinyl-1,2,4-triazolium iodide) against time.

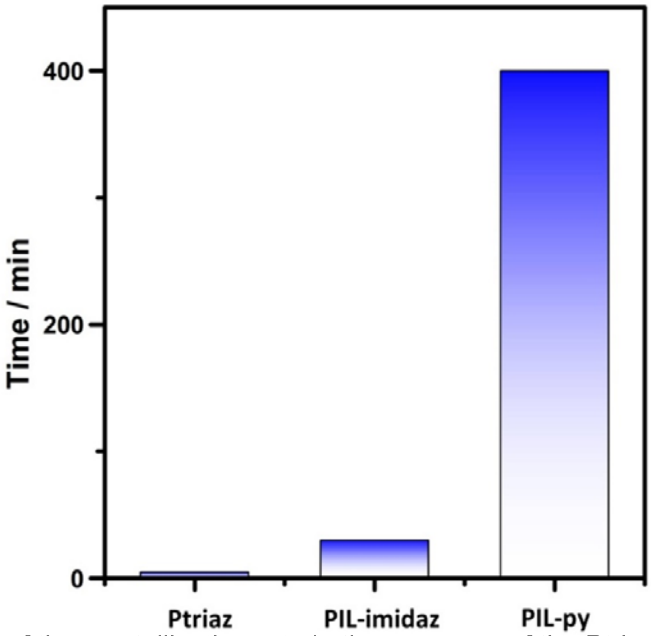

Figure S18. Comparison of the crystallization rate in the presence of the Ptriaz, PIL-imidaz, PIL-py.

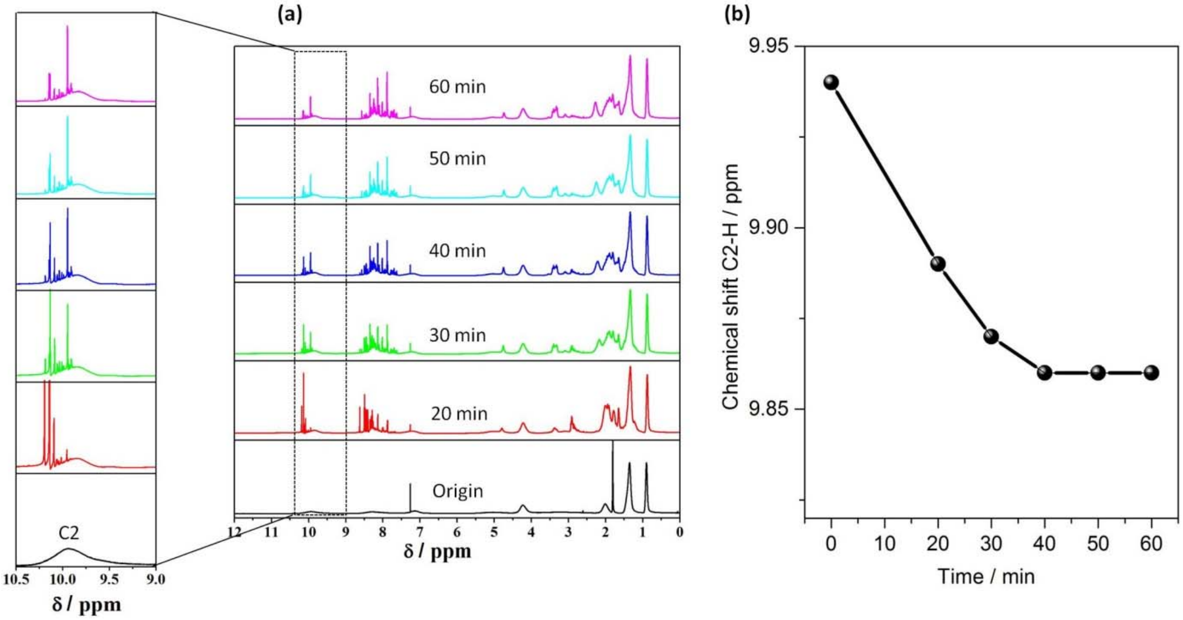

Figure S19. a, In situ ^{1}H NMR spectra of the CC3R synthesis mixtures in PIL-imidaz in deuterated chloroform at room temperature. The corresponding C2-proton in PIL-imidaz during the CC3R synthesis with higher magnification was highlighted in left column. b, Chemical shift (dark line) of C2- proton peaks in PIL-imidaz against time (The degree of chemical shift is much less than of C5-proton in Ptriaz). The integration of C2-proton is skipped due to overlap with precursors of CC3R.

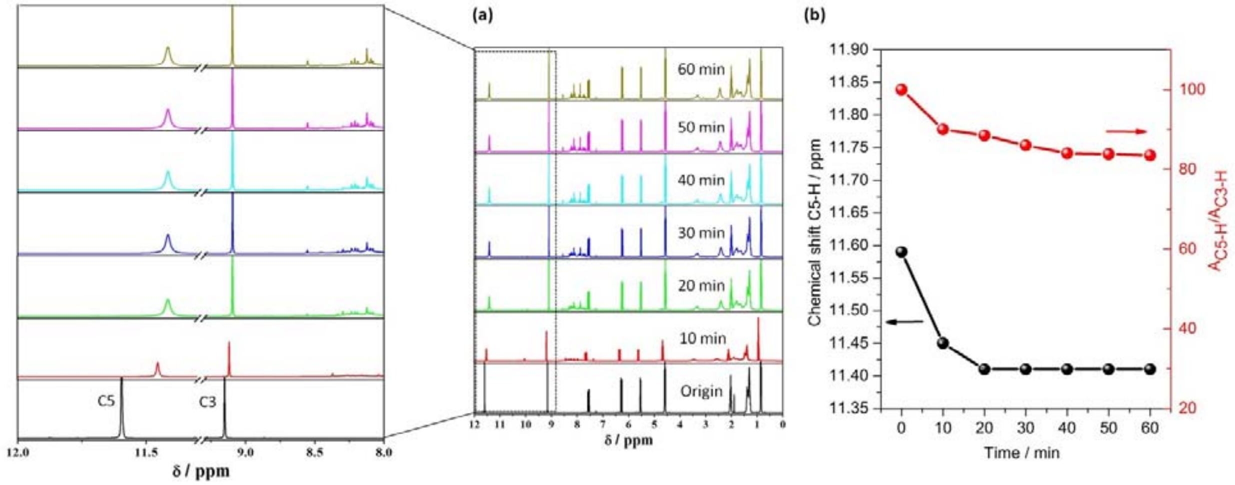

Figure S20. a, In situ ^{1}H NMR spectra of the CC3R synthesis mixtures in triaz monomer in deuterated chloroform at room temperature. The corresponding C5- and C3-protons in triaz monomer during the CC3R synthesis with higher magnification was highlighted in left column. b, Chemical shift (dark line) and integration of C5- proton peaks (red line) in triaz against time.

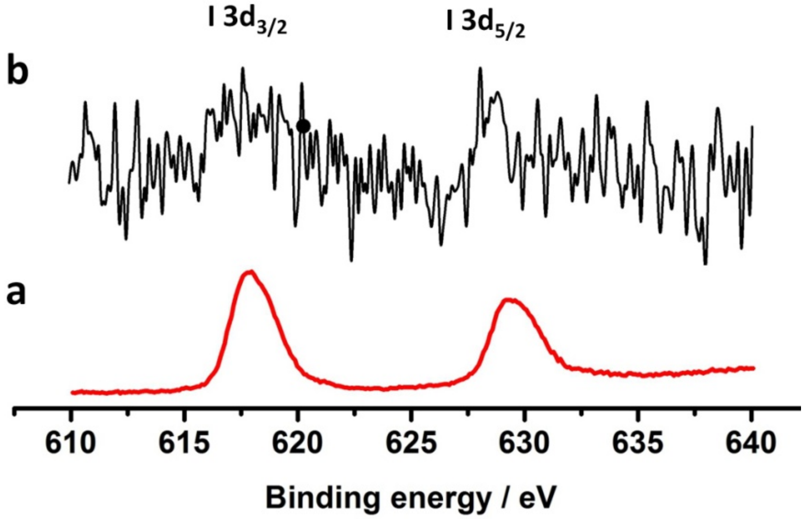

Figure S21. The XPS spectra for I 3d signals of Ptriaz (a) and CC3R-Ptriaz (b).

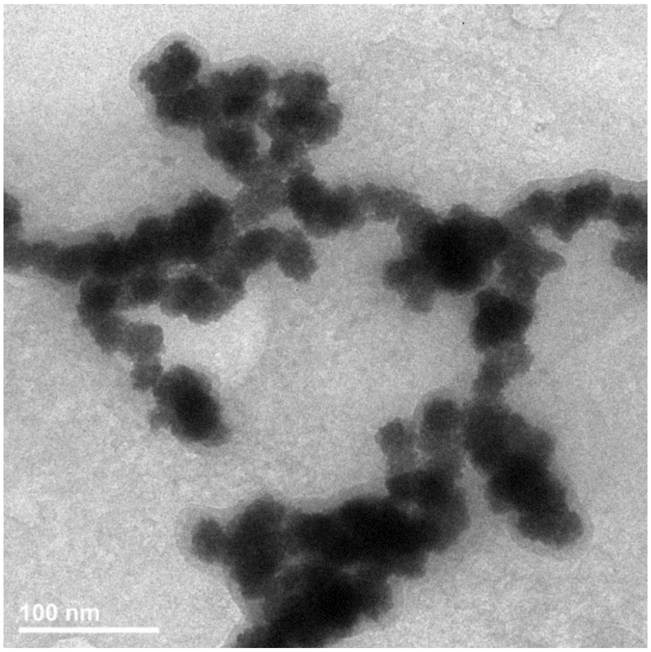

Figure S22. The bright field (BF) TEM image of Rh-SP-Free catalyst.

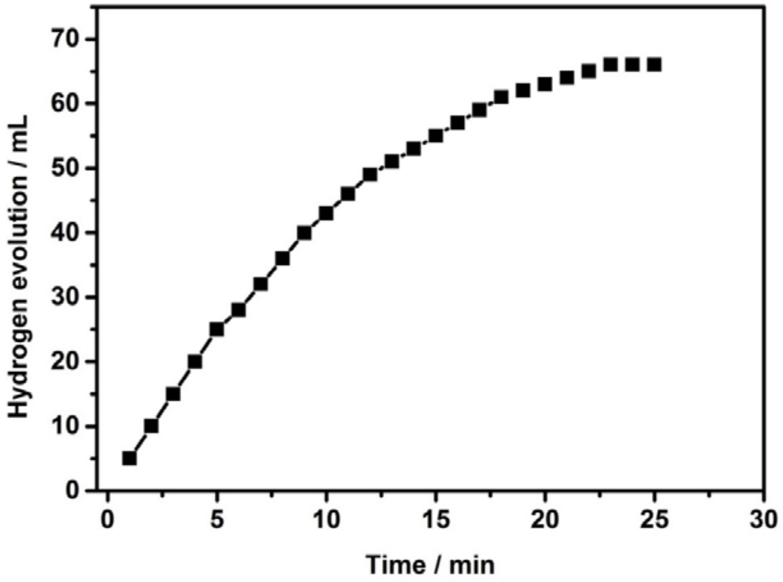

Figure S23. Time course plots of H_2 generation for the methanolysis of ammonia borane (AB) over the Rh-SP-Free catalyst at 298 K (Rh/AB = 0.01).

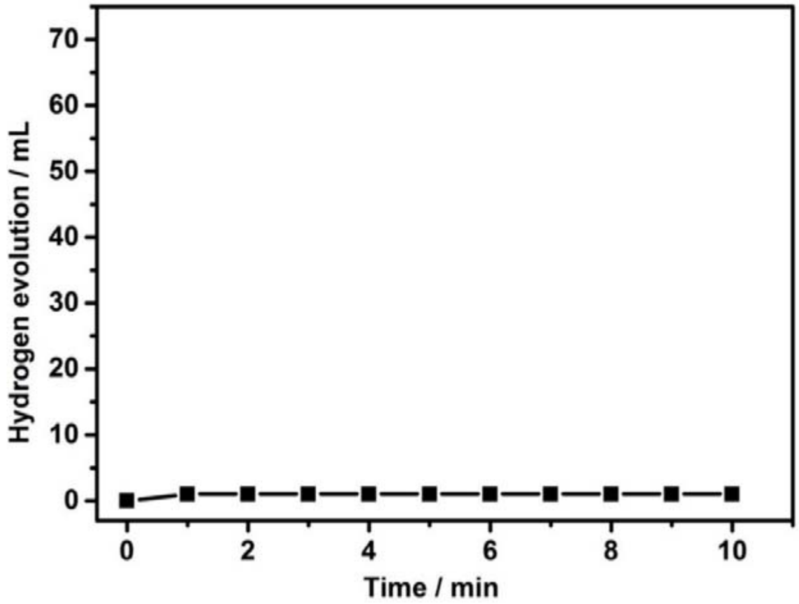

Figure S24. Time course plots of H_2 generation for the methanolysis of AB over the Ptriaz at 298 K.

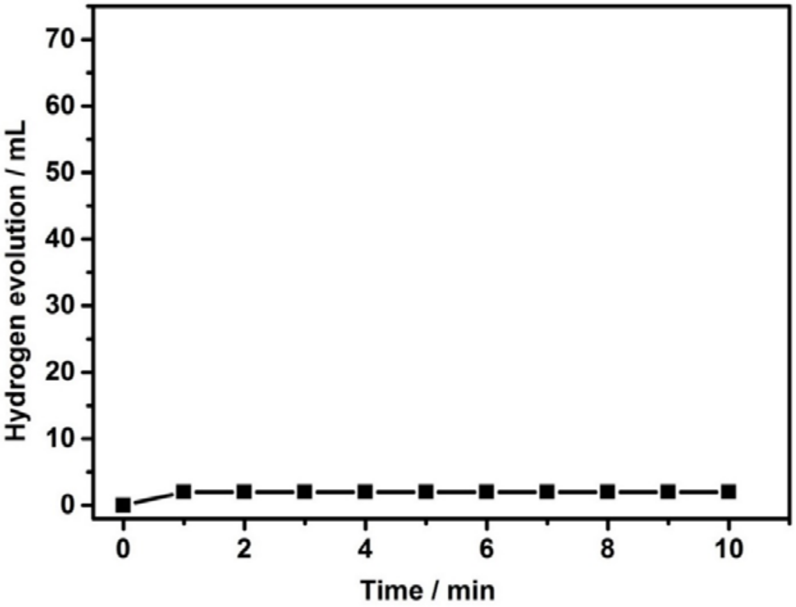

Figure S25. Time course plots of H_2 generation for the methanolysis of AB over the CC3R at 298 K.

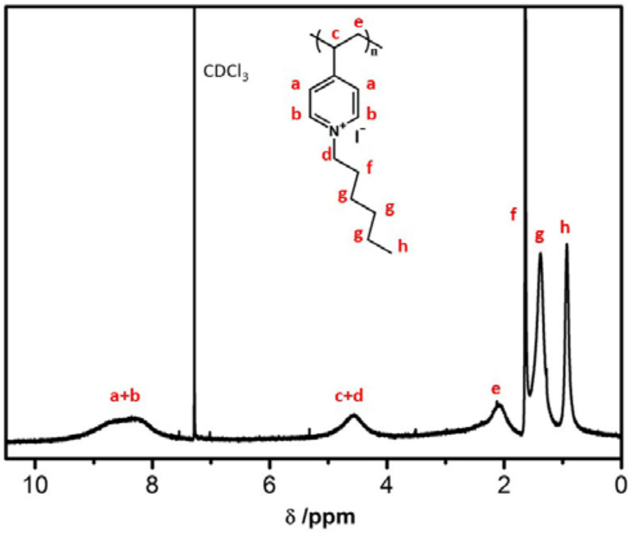

Figure S26. Chemical structure and ^{1}H-NMR spectrum of PIL-py.

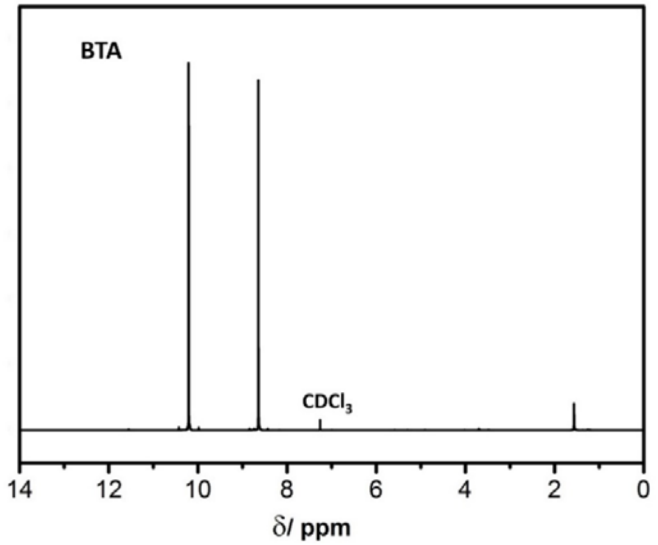

Figure S27. The ^{1}H-NMR spectrum of BTA in $CDCl_3$.

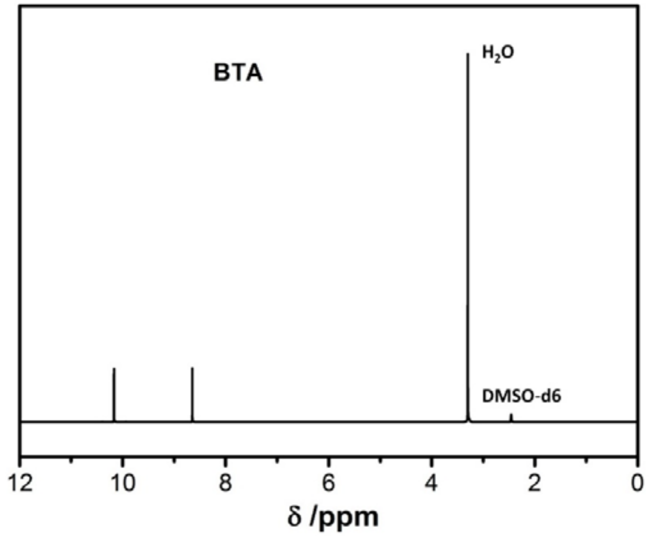

Figure S28. The ^{1}H-NMR spectrum of BTA in DMSO-d6.

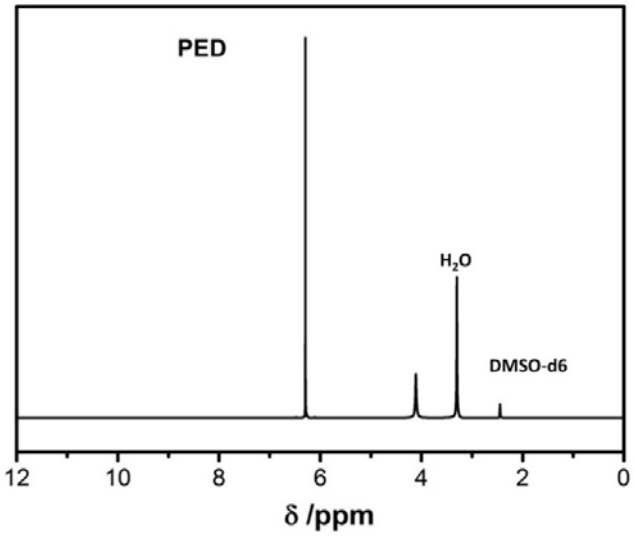

Figure S29. The ^{1}H-NMR spectrum of PED in DMSO-d6.

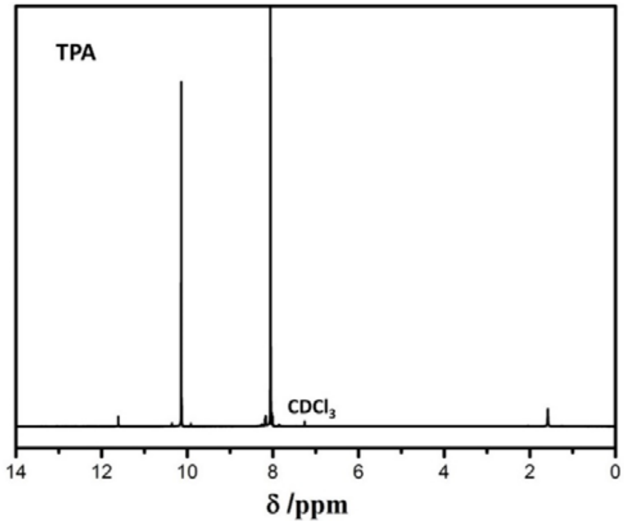

Figure S30. The ^{1}H-NMR spectrum of TPA in $CDCl_3$.

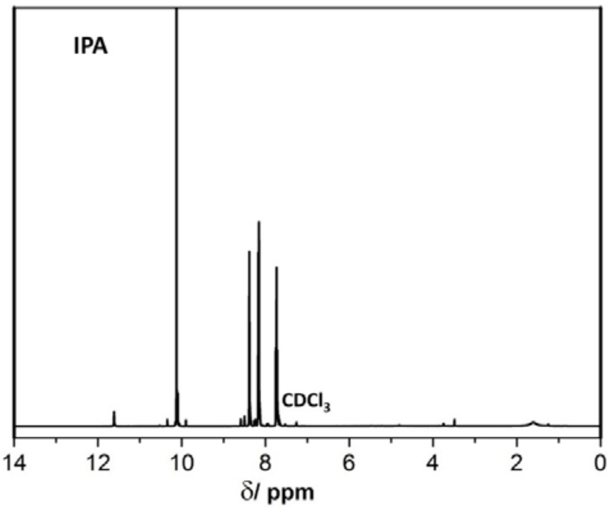

Figure S31. The ^{1}H-NMR spectrum of IPA in $CDCl_3$.

4. Computational details for imine bond formation reaction

Density functional theory (DFT) calculations were carried out to understand the reaction mechanism between 1,2-Diaminocyclohexane and 1,3,5-Benzenetricarboxaldehyde in the presence of triazolium catalyst (the triazolium monomer was used for calculation) by using Gaussian 09 software package[1]. Geometry optimization was carried out at the M062x method[2] with a 6-31+G(d,p) basis set[3] and in implicit solvent chloroform using PCM as solvation model[4] as implemented in Gaussian 09. The description of van der Waals interactions was improved using Grimme's empirical dispersion (GD3) correction[5]. Frequency calculations, at the same level of theory, were used to obtain thermal corrections (at 298 K) and to characterize optimized structures as transition states (only a single imaginary frequency) or intermediate (if no imaginary frequencies were found). Single point energy calculations were performed at the M06-2x/Def2-TZVP level.[6] The Intrinsic reaction coordinate (IRC)[7] calculations of transition states were performed to confirm the appropriate connected reactants and products.

The interaction between 1,2-Diaminocyclohexane and triazolium ring

In the experiment, the up-field shift of the C5-proton signal was observed (Figure S12) when mixing Ptriaz with 1,2-Diaminocyclohexane because of the hydrogen bonding (C5-H…N) interaction between -NH_2 and triazolium moiety, which have been also revealed by theoretical calculation, an increase of C-H from 1.079 (triazolium) to 1.097 Å (triazolium + 1,2-Diaminocyclohexane) could be observed (Figure S32). Such strong interaction between 1,2-Diaminocyclohexane and Ptriaz favorably impair the strength of the C-H bond, leading to easier ionization of the C5-proton and enhancing acidity that catalyzes the imine bond formation reaction (as evidenced by in situ ^{1}H NMR spectra of CC3R formation process).

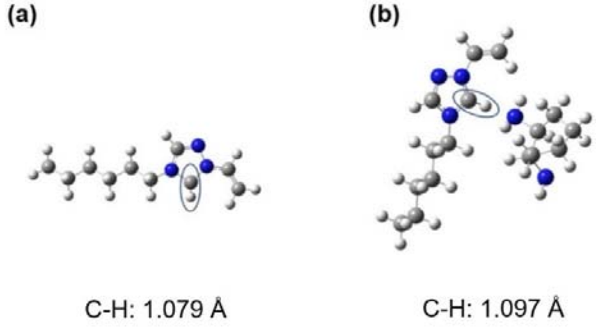

Figure S32. (a) The C5-H distance of (a) Ptriaz, (b) Ptriaz in the presence of 1,2-Diaminocyclohexane (White, H; gray, C; red, O; blue, N).

References

[1]. M. J. Frisch, G. W. Trucks, H. B. Schlegel, G. E. Scuseria, M. A. Robb, J. R. Cheeseman, G. Scalmani, V. Barone, B. Mennucci, G. A. Petersson, H. Nakatsuji, M. Caricato, X. Li, H. P. Hratchian, A. F. Izmaylov, J. Bloino, G. Zheng, J. L. Sonnenberg, M. Hada, M. Ehara, K. Toyota, R. Fukuda, J. Hasegawa, M. Ishida, T. Nakajima, Y. Honda, O. Kitao, H. Nakai, T. Vreven, J. A. Montgomery, Jr., J. E. Peralta, F. Ogliaro, M. Bearpark, J. J. Heyd, E. Brothers, K. N. Kudin, V. N. Staroverov, R. Kobayashi,

J. Normand, K. Raghavachari, A. Rendell, J. C. Burant, S. S. Iyengar, J. Tomasi, M. Cossi, N. Rega, J. M. Millam, M. Klene, J. E. Knox, J. B. Cross, V. Bakken, C. Adamo, J. Jaramillo, R. Gomperts, R. E. Stratmann, O. Yazyev, A. J. Austin, R. Cammi, C. Pomelli, J. W. Ochterski, R. L. Martin, K. Morokuma, V. G. Zakrzewski, G. A. Voth, P. Salvador, J. J. Dannenberg, S. Dapprich, A. D. Daniels, Ö. Farkas, J. B. Foresman, J. V. Ortiz, J. Cioslowski, and D. J. Fox, Gaussian, Inc., Wallingford CT, **2009**.

[2]. Y. Zhao, D. G. Truhlar, *Theor.Chem. Acc.* **2008**.

[3]. W. J. Hehre, R. Ditchfield, J. A. Pople, *J. Chem. Phys.* **1971,** *54*, 724-728.

[4]. J. Tomasi, B. Mennucci, R. Cammi, *Cheminform* **2005,** *105*, 2999-3093.

[5]. S. Grimme, J. Antony, S. Ehrlich, H. Krieg, *J. Chem. Phys.* **2010,** *132*, 154104-0.

[6]. A. Schäfer, C. Huber, R. Ahlrichs, *J. Chem. Phys.* **1994,** *100*, 5829-5835.

[7]. K. Fukui, *Acc. Chem. Res.* **1981,** *14*, 363-368.
